# Electric Field-Modulated Electrospray Ionization Mass Spectrometry for Quantity Calibration and Mass Tracking

**DOI:** 10.1021/jasms.4c00091

**Published:** 2024-05-24

**Authors:** Pin-Chieh Hsu, Pawel L. Urban

**Affiliations:** Department of Chemistry, National Tsing Hua University, 101, Section 2, Kuang-Fu Road, Hsinchu 300044, Taiwan

## Abstract

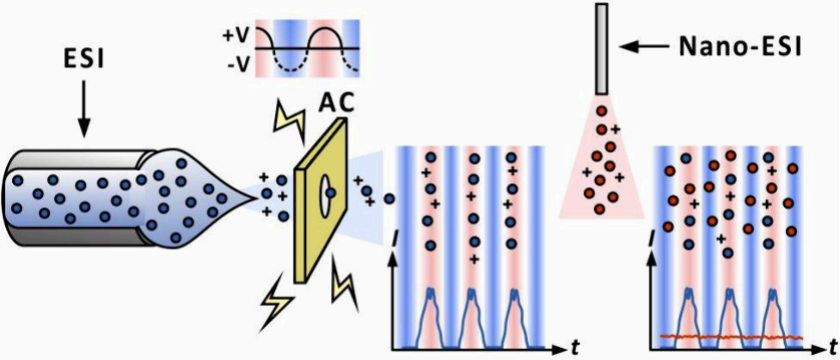

Analyses conducted by electrospray ionization (ESI) mass spectrometry (MS) typically entail performing a number of preparatory steps, which include quantity calibration and mass calibration. Quantity calibration can be affected by signal noise, while mass calibration can be affected by instrumental drift if analyses are performed over an extended period of time. Here, we present two methods for achieving these calibrations using modulation of electrospray plume by alternating electric fields and demodulating the resulting MS ion currents. For this purpose, we use an ESI source fitted with three ring electrodes between the electrospray emitter and the mass spectrometer’s inlet. One of these electrodes is supplied with a sine electric signal. Optionally, a nanoESI emitter is also placed between the ring electrodes and the mass spectrometer’s orifice to supply calibrant ions. The ion currents, recorded with this setup, present wave-like features. In the first variant, using a triple quadrupole mass analyzer, the ion currents are subjected to data treatment by fast Fourier transform (FFT), and the resulting FFT magnitudes are correlated with analyte concentrations to produce a calibration plot. In the second variant, using a quadrupole time-of-flight mass analyzer, the mass spectra recorded at the analyte ion current maxima are mass-checked using the *m*/*z* value of the internal standard (injected via nanoESI emitter), which appears predominantly in the time intervals corresponding to the analyte ion current minima. The setup has been characterized using simulation software and optimized. Overall, the method enables the preparation of quantity calibration plots and monitoring (minor) *m*/*z* drifts during prolonged analyses.

## Introduction

Electrospray ionization (ESI) is a popular soft ionization source for producing gaseous ions prior to mass spectrometry (MS).^1−4^ Its outstanding features include high compatibility with liquid chromatography (LC), but also suitability for analysis of thermally unstable substances, such as proteins.^5−7^ However, several factors can indirectly cause variability of the instrumental response, leading to low reproducibility.^8,9^ Notably, among these factors are matrix effects, fluctuations in LC performance, variations in the ESI current, and variations in detector sensitivity.^8,10−12^ Therefore, to compensate for the inherent variation of instrument signal in analysis, addition of calibration standard is the optimal method to improve reproducibility in MS.^9^

External calibration is a commonly used method for quantitative analysis. By establishing a linear relationship between instrument response and concentration, the concentration of the target analyte can be calculated. Additionally, this method also allows for mass calibration, which ensures accurate mass measurement of the sample ions by introducing a reference compound with a known mass-to-charge ratio (*m*/*z*) into the mass analyzer.^13^ It is less restrictive than internal calibration and addresses the issues of ion suppression and matrix effects encountered in internal calibration.^13^ Nonetheless, because mass calibration is typically conducted before analysis, the calibrant ions may not experience conditions, such as acceleration voltages or space charge effects, precisely identical to those observed in the case of analytes.^13^ Consequently, external calibration is prone to instrumental drifts. These drifts may be caused by humidity, temperature, or external magnetic field.^14−16^ To address the problem of the time-dependent instrumentation drift in external calibration, many studies have employed a dual-electrospray ion source arrangement with secondary spray for calibration, which is available in some modern time-of-flight (TOF) instruments (e.g., refs (13) and (17−19)).

The dual-electrospray calibration technique aims to generate the separate signal of sample and reference sprays in sequence by either alternately switching the high voltage between the two sprayers^20,21^ or physically blocking one of the sprayers.^19,22−25^ This design requires sacrificing a short analysis time to obtain calibration spectra.^13^ However, it provides the flexibility to adjust the sampling time of either the calibrant or the analyte, as well as the option to use different solvent systems, flow rates, and electrospray potentials.^19^ Consequently, the dual-electrospray ionization source, which introduces the analytes and calibrants via two injectors, has emerged as a promising technique to effectively address the concerns associated with preferential ionization and suppression effects arising from the mixing of analytes and calibrants in internal calibrations.^19,22,26^ It has also been reported that placing two nanosprays in the same AC electric field can enable internal calibration. In this case, the induced nanospray technique mitigates repulsions between analyte ions and calibrant ions.^27^

One of the objectives in analytical method development is the enhancement of the signal-to-noise ratio (S/N). For that purpose, different data processing methods have been employed, which include Fourier transform, discrete Fourier transform (DFT), and Hadamard transform (HT).^28−30^ For example, extra-column dispersion in chromatography can be addressed using DFT to eliminate periodic components and reduce high-frequency random noise in analytical signals, enhancing the S/N and significantly increasing the number of theoretical plates. This also improves peak resolution, identification, and quantification accuracy in single and multidimensional separation techniques.^31^ In addition, Cheng et al. employed the HT technique, in combination with gas chromatography, to achieve rapid and sensitive online detection of low-concentration samples, without the need for additional extraction steps.^32^ Another strategy to improve analytical performance and enable multiplexing is to introduce samples with specific signals or features to generate periodic signals that can be processed in the frequency domain, and then demodulated by a mathematical treatment.^33,34^ For example, Allen et al. used microfluidic chips to produce a series of linearly spaced peaks for each analyte in the sample with a periodicity determined by its mobility.^35^ Alternatively, one can inject multiple samples into a single flow line, at unique frequencies, to multiplex MS analyses.^36^

In this study, we aimed to develop an approach for the quantitative ESI–MS analysis of samples using electrospray plume modulation and subsequent fast Fourier transform (FFT) signal demodulation. Additionally, we demonstrate a variant of the proposed device with the addition of a nanospray emitter to achieve mass tracking, which may address the unavoidable signal drift problem associated with external calibration.

## Experimental Section

### Chemicals

Methanol (LC–MS grade) and water (LC–MS grade) were purchased from Merck (Darmstadt, Germany). Acetaminophen (98.0–102.0%, meets USP testing specs), adipic acid (99%), l-glutamine (≥99%, reagent grade), and lysine (≥98.0%, TLC) were purchased from Sigma-Aldrich (St. Louis, MO). l-Alanine (99%, nitrogen flushed) was purchased from Acros Organics (Geel, Belgium).

### Experimental Setup for Quantitative Analysis by QQQ-MS

**Figure 1:**
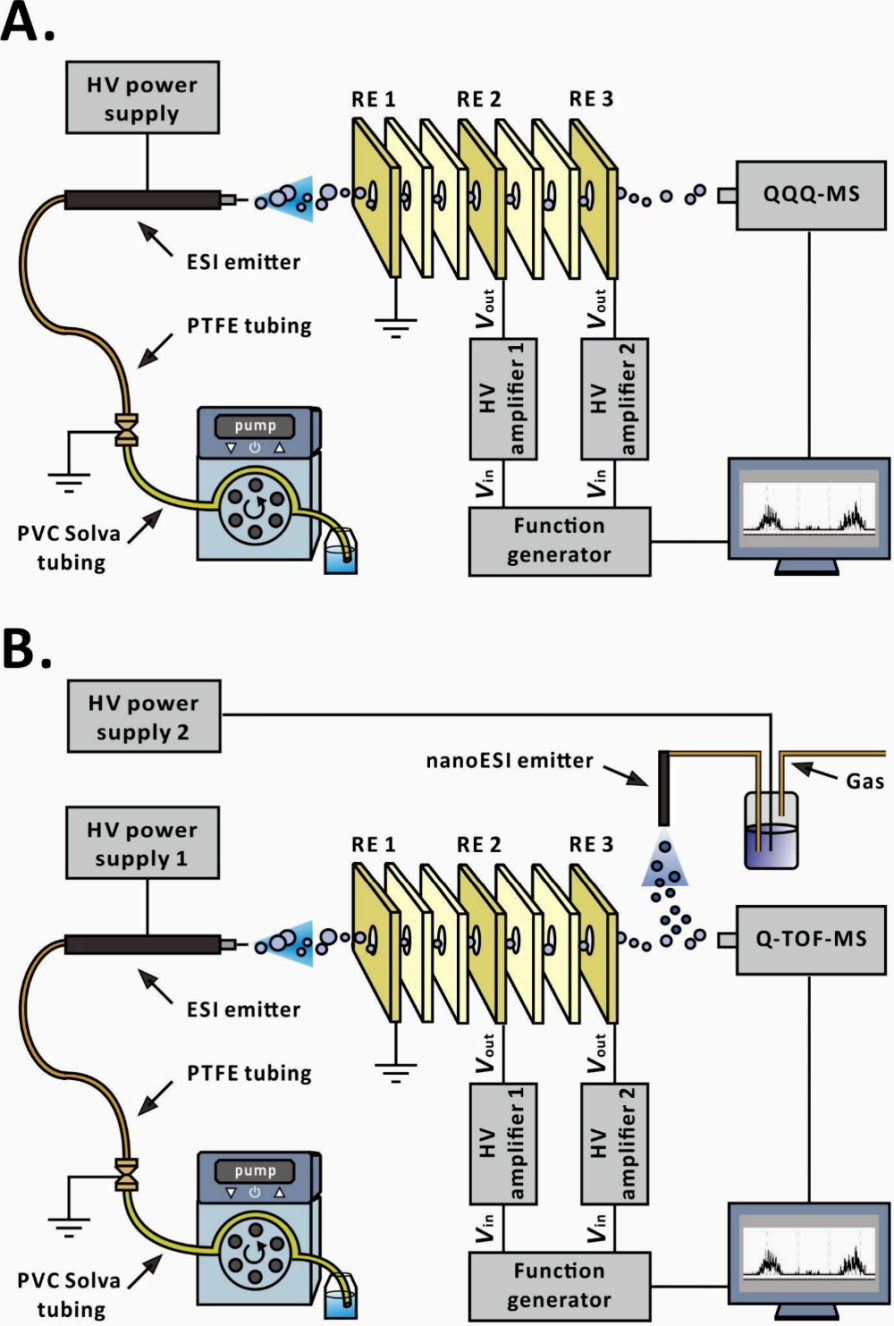
Simplified scheme of the experimental setup: (A) QQQ-MS quantification experiment and (B) Q-TOF-MS qualification experiment.

A house-built ESI system, incorporating an ESI capillary (ID, 0.1 mm; OD, 0.27 mm; length, 82.5 mm; part no. 225-14915; Shimadzu, Kyoto, Japan), was fixed in a 3D-printed holder (core material, acrylonitrile-butadiene-styrene; Tiertime, Beijing, China), which was set up in front of a triple-quadrupole mass spectrometer (QQQ-MS; LCMS-8030; Shimadzu; Figure 1A). Positive-ion selected-ion monitoring (SIM) mode was employed. The nitrogen drying gas flow rate was set to 3.0 L min^–1^, while its temperature was 400 °C. The temperature of the desolvation line (DL) was set to 150 °C. To avoid the drying gas flow blowing directly into the electrospray plume, which might cause instability in the MS signal, the vertical position of the ESI capillary was higher than the MS inlet by ∼1 mm (Figure S1). Between the ESI capillary and the MS inlet, three ring electrodes (REs) with different opening diameters (width, 40 mm; length, 40 mm; thickness, 0.8 mm; part no. CGS-1015-0.8-single; material, glass fiber with copper coated on one side; Centenary Materials, Hsinchu, Taiwan) were arranged coaxially in a stack, as described in the previous work.^37^ The opening diameters of the RE1, RE2, and RE3 were 10, 5, and 7.5 mm, respectively. Two insulation plates with central opening [diameter, 25 mm; width, 40 mm; length, 40 mm; thickness, 1 mm; part no. 130680; material, polytetrafluoroethylene (PTFE); Centenary Materials] were placed between the adjacent REs. The distance between the ESI capillary and the RE1 was ∼7 mm, while the distance between the RE3 and the sampling cone was ∼12.5 mm. The test sample was pumped by a peristaltic pump at 40 μL min^–1^ (model no. MF-10; Yotec Precision Instrument, Hsinchu, Taiwan), transferred through PVC Solva tubing (168-001A-101; ID, 0.13 mm; OD, 2.05 mm; length, 420 mm; Ismatec), a section of PTFE tubing (ID, 0.3 mm; OD, 1.59 mm; length, 70 mm; part no. 58702; Supelco, Merck, Darmstadt, Germany), via a grounded metal union (part no. U-438; IDEX Health & Science, Rohnert Park, CA), and another section of PTFE tubing (ID, 0.3 mm; OD, 1.59 mm; length, 300 mm; part no. 58702; Supelco) to the ESI capillary.

A high-voltage (HV) power supply 1 (MPS10P10; Spellman, Hauppauge, NY) was used to supply 4.0 kV to the ESI capillary (Figure 1A). The RE1 was grounded. The RE2 and RE3 were supplied with voltages controlled by the function generator (Analog Discovery 2, AD2, part no. 210-321; Digilent, Pullman, WA; Figure S2). Through program control, a sine waveform (*V*_pp_ = 0.4 V) was output to the input of HV amplifier 1 (gain = 500; TREK 609A-1; Advanced Energy, Denver, CO) by W1 channel, providing alternating current (AC) signal of 200 V to the RE2, while a direct current (DC, amplitude = 0.1 V) was output to the input of HV amplifier 2 (gain = 100; 2350S-100-2K; TEGAM, Geneva, OH) by W2 channel, providing 10 V DC to the RE3. To trigger the mass spectrometer, and start the data acquisition, a relay board (model no. 2R1B; Centenary Materials) was powered with 5 V, and its input was connected to pin 6 of the Analog Discovery 2 module to receive a trigger signal.

### Experimental Setup for Qualitative Analysis by Q-TOF-MS

A house-built ESI system, incorporating an ESI capillary (ID, 0.1 mm; OD, 0.27 mm; length, 82.5 mm; part no. 225-14915; Shimadzu), was fixed in a 3D-printed holder (core material, acrylonitrile-butadiene-styrene; Tiertime), which was set up in front of a Q-TOF mass spectrometer (Q-TOF-MS; LCMS-9030; Shimadzu; Figure 1B). The mass analyzer was operated in the MS scan mode. The ion source chamber of the Q-TOF-MS instrument was replaced with a house-built source containing both ESI and nanoESI emitters. The flow rate of drying gas (nitrogen) was 3.0 L min^–1^, while the temperature of the drying gas was 400 °C. The temperature of the DL was 200 °C. To avoid the drying gas flow blowing directly into the electrospray plume, which might cause instability of the MS signal, the vertical position of the ESI capillary was higher than that of the MS inlet by ∼1 mm. Between the ESI capillary and the MS inlet, three REs with different opening diameters (width, 40 mm; length, 40 mm; thickness, 0.8 mm; part no. CGS-1015-0.8-single; material, glass fiber with copper coated on one side; Centenary Materials) were arranged coaxially in a stack, as described in the previous work.^37^ The opening diameters of the RE1, RE2, and RE3 were 10, 5, and 7.5 mm, respectively. Two insulation plates with central opening (diameter, 25 mm; width, 40 mm; length, 40 mm; thickness, 1 mm; part no. 130680; material, PTFE; Centenary Materials) were placed between the adjacent REs. The analyte sample was pumped by a peristaltic pump at 10 μL min^–1^ (model no. MF-10; Yotec Precision Instrument), transferred through PVC Solva tubing (168-001A-101; ID, 0.13 mm; OD, 2.05 mm; length, 420 mm; Ismatec), a section of PTFE tubing (ID, 0.3 mm; OD, 1.59 mm; length, 70 mm; part no. 58702; Supelco), via a grounded metal union (part no. U-438; IDEX Health & Science), and another section of PTFE tubing (ID, 0.3 mm; OD, 1.59 mm; length, 300 mm; part no. 58702; Supelco) to the ESI capillary. The distance between the ESI capillary and the RE1 was ∼7 mm, while the distance between the RE3 and the sampling cone was 10 mm. The nanoESI capillary was fixed to a ceramic rod (ID, 1 mm; OD, 4 mm; length, 18 mm) within the 3D-printed holder (core material, acrylonitrile-butadiene-styrene; Tiertime), which was placed between the RE3 and the sampling cone. The internal standard was placed in a container with a high-voltage wire and gas tubing, transferred through a capillary tubing section (ID, 0.05 mm; OD, 0.38 mm; length, 450 mm; part no. 1010-31845; GL Sciences, Tokyo, Japan), which was connected to another section of the capillary tubing (ID, 0.02 mm; OD, 0.38 mm; length, 150 mm; part no. 1010-31445; GL Sciences) via a PTFE tubing section (ID, 0.38 mm; OD, 0.84 mm; length, 20 mm).

A high-voltage (HV) power supply 1 (MPS10P10; Spellman) applied 4.0 kV to the ESI capillary, while the HV power supply 2 (MPS10P10; Spellman) applied 4.0 kV to the nanoESI electrolyte solution vial (Figure 1B). The RE circuit and the trigger of the mass spectrometer were the same as in the QQQ-MS part.

### QQQ-MS Data Treatment

Ion current raw data for selected ions were exported to ASCII files using the LabSolutions software (version 5.97; Shimadzu). Subsequently, these ASCII files were imported into Excel software (version 2019; Microsoft, Redmond, WA) to compute the average signal intensity and standard deviations for each concentration without AC treatment (in three replicates). For the portion with AC treatment, FFT computations were performed using Matlab (version R2021a; MathWorks, Natick, MA). The FFT magnitude was determined for each concentration (in three replicates) at a frequency of 0.0416 Hz, corresponding to the pattern observed in the raw data of QQQ-MS ion currents. The FFT magnitudes for each concentration were then imported to Excel software to calculate the average signal intensities and standard deviations. Finally, the results for both data sets, with and without AC treatment, were plotted using OriginPro software (version 8.5; OriginLab, Northampton, MA).

### Q-TOF-MS Data Treatment

In the 8 h temperature elevation experiment, we used the LabSolutions software (version 5.113; Shimadzu) to average the data and record the *m*/*z* measurements of samples and calibrants every 20 min. The mass error (*e*_m_ [ppm]) of adipic acid, without correction by glutamine, was calculated using the equation:
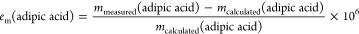
1where *m*_measured_ is the measured *m*/*z*, and *m*_calculated_ is the calculated *m*/*z*.

The mass error of adipic acid corrected by glutamine (*e*_m-corrected_ [ppm]) was calculated using the equation:
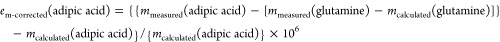
2

Finally, the results for both data sets, with and without calibrant correction, were plotted using the OriginPro software to show the trends of the mass error as it varied over time in the environment affected by temperature change.

### Calculation of Mass and Charge of Microdroplets for Simulations

In the COMSOL Multiphysics (version 6.2; COMSOL, Burlington, MA) simulation, we set the boundary with a width of 42 mm and a height of 33.5 mm. Inside this boundary, three components were included (Figure 1A): the ESI capillary, REs, and MS inlet. The boundary conditions were as follows: a voltage of 4.0 kV was applied to the ESI capillary; the space between the ESI capillary and the MS inlet was filled with air; and the relative permittivity of the air was 1. After the model was built, the electric potential and electric field streamline were chosen to present the results. To understand the trajectories of charged droplets in the presence of an electric field, we simulated motion of droplets with various diameters using SIMION (version 2020-07-01-8.2.0.5; Scientific Instrument Services, Palmer, MA). In the case of the SIMION simulation, a voltage of 4.0 kV was applied to the ESI capillary. The mass and diameter of the collision gas were ∼29 amu and 0.366 nm, respectively, while the pressure was 760 Torr. The three simulated droplet diameters were 1 nm, 1 μm, and 100 μm, respectively. We assumed that each particle is spherical and calculated their volumes (*V*) using the formula:
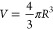
3where *V* is the volume of particle (m^3^) and *R* is the radius of droplet (m). Because the sample solvent was 25% (v/v) MeOH, we considered densities (*D*) of methanol and water (997.8 and 791.4 kg m^–3^, respectively) at room temperature (298.15 K). Subsequently, we determined the mass distribution limits for each diameter:
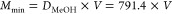
4
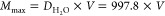
5

Particle charges at Rayleigh limit (*Q*_R_) were calculated based on the equation:^38^
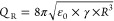
6where ε_0_ is the electrical permittivity of vacuum and γ is the surface tension. We then determined the charge distribution limits for each diameter:
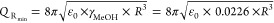
7
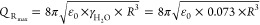
8In all of the trajectory simulations, a total of 200 particles were tested. Considering the potential differences in density and surface tension of water and methanol within the droplets, we utilized a uniform distribution to simulate the mass and charge of the 200 particles.

## Results and Discussion

### Numerical Simulations of the ESI System with Three Ring Electrodes

**Figure 2:**
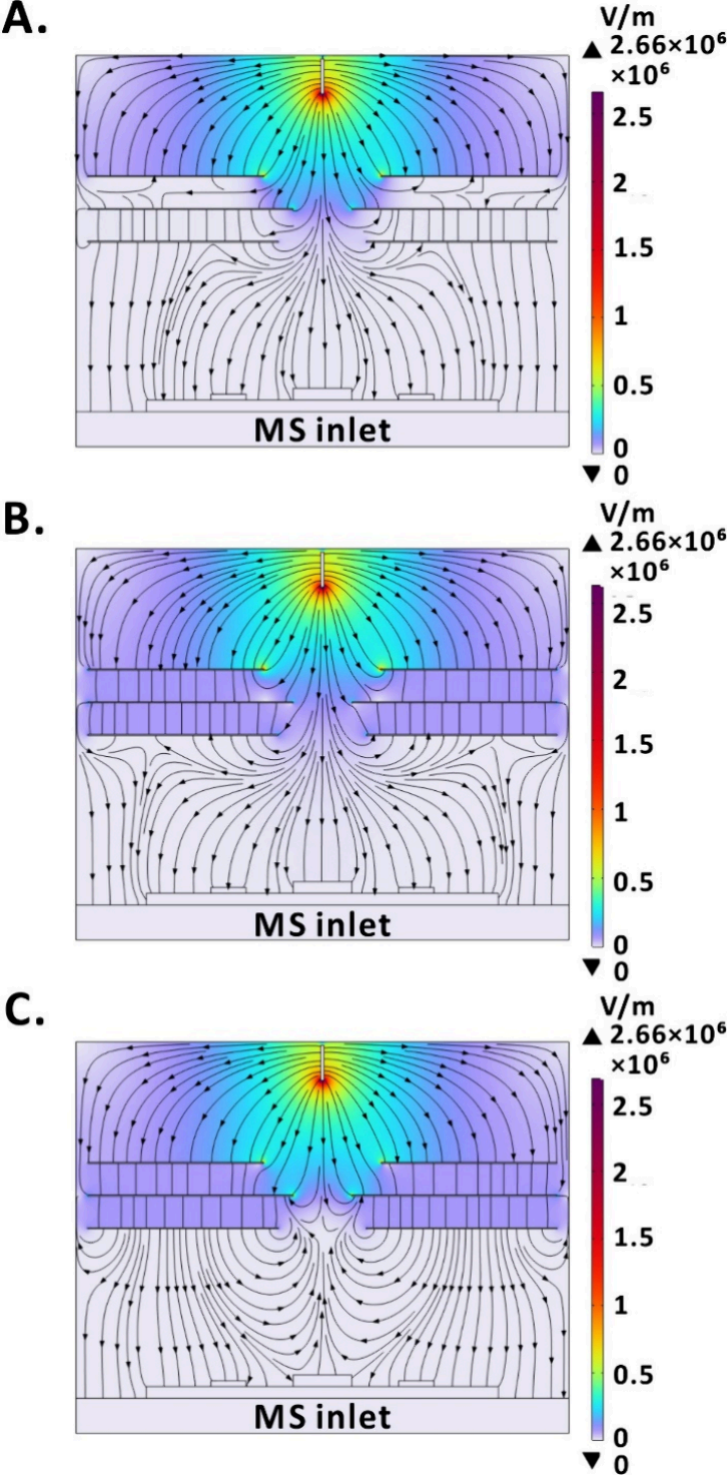
COMSOL simulation of the electric field: (A) when the AC voltage on the RE2 crosses 0 V; (B) when the AC voltage on the RE2 crosses 200 V; and (C) when the AC voltage on the RE2 crosses −200 V. Color contours: equipotential zones. Black lines with arrows: electric field streamlines.

**Figure 3:**
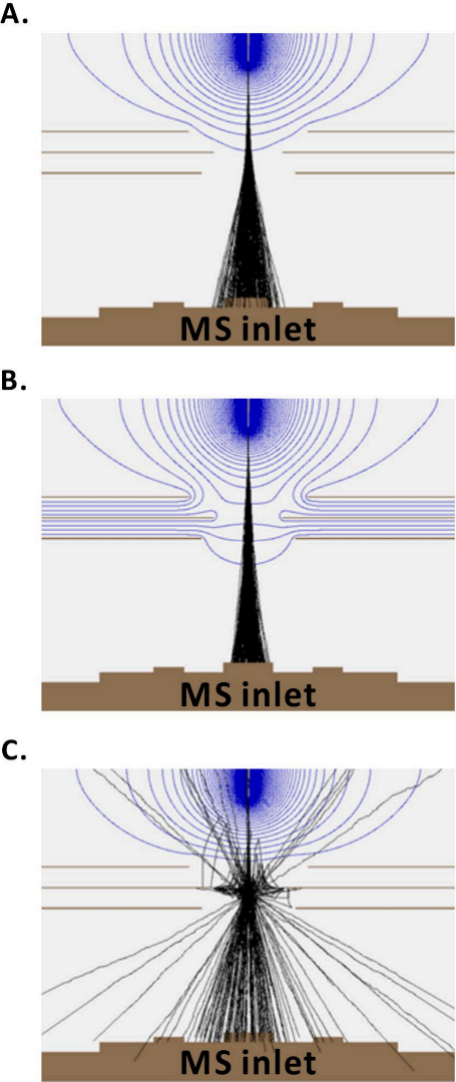
SIMION simulation of the particles with diameter 1 μm (*Q*_min_ ≈ 2.48 × 10^4^ e, *Q*_max_ ≈ 4.46 × 10^4^ e and *M*_min_ ≈ 2.50 × 10^11^ u, *M*_max_ ≈ 3.15 × 10^11^ u): (A) when the AC voltage on the RE2 crosses 0 V; (B) when the AC voltage on the RE2 crosses 200 V; and (C) when the AC voltage on the RE2 crosses −200 V. Blue lines: equipotential lines. Black lines: trajectories of particles. *Q*, particle charge; *M*, particle mass.

To investigate the impact of the AC field on charged droplets, as they pass through the REs, we used both COMSOL and SIMION software. The COMSOL software provided detailed electric field-related data such as the convergence of electric field streamlines. The SIMION software, which simulates charged particle (ion) motion, was used to model the trajectories of charged particles under the electric field. By comparing the results from both simulation software packages, one can make more accurate conjectures about the actual trajectories of particles. These simulations aimed to verify the trajectories of the droplets before entering the MS inlet under varying electric field conditions. Both simulation results are depicted in 2D graphs (Figures 2 and 3). In these simulations, the RE1 was grounded, the RE3 was maintained at a constant DC potential of 10 V, while the RE2 was supplied with three voltages: (A) 0 V (Figures 2A and 3A); (B) 200 V (Figures 2B and 3B); and (C) −200 V (Figures 2C and 3C).

Based on the COMSOL simulation results, it is evident that, when the AC voltage, applied to the RE2, passes through 0 V (Figure 2A) and −200 V (Figure 2C), a strong electric field emerges at the edge of the RE2 hole (indicated with the light blue color). This suggests that positively charged analytes may be retarded before entering the MS inlet. Particularly, when the RE2 is supplied with −200 V (Figure 2C), the black lines with arrows, representing the electric field streamlines, point in the opposite direction with respect to the MS inlet. This retarding force decreases the probability of ions and charged droplets entering the MS inlet. However, when the voltage, applied to the RE2, reaches 200 V (Figure 2B), the attenuation of the strong electric field near the edge of the RE2 hole occurs. Because the retarding force, generated by the RE2, is diminished, droplets and ions can be guided toward the MS inlet. It is also worth noting that a weak electric field is present near the edge of the RE3 hole (Figure 2B), which might generate an additional thrust to assist positively charged ions and droplets in entering the MS inlet.

**Figure 4:**
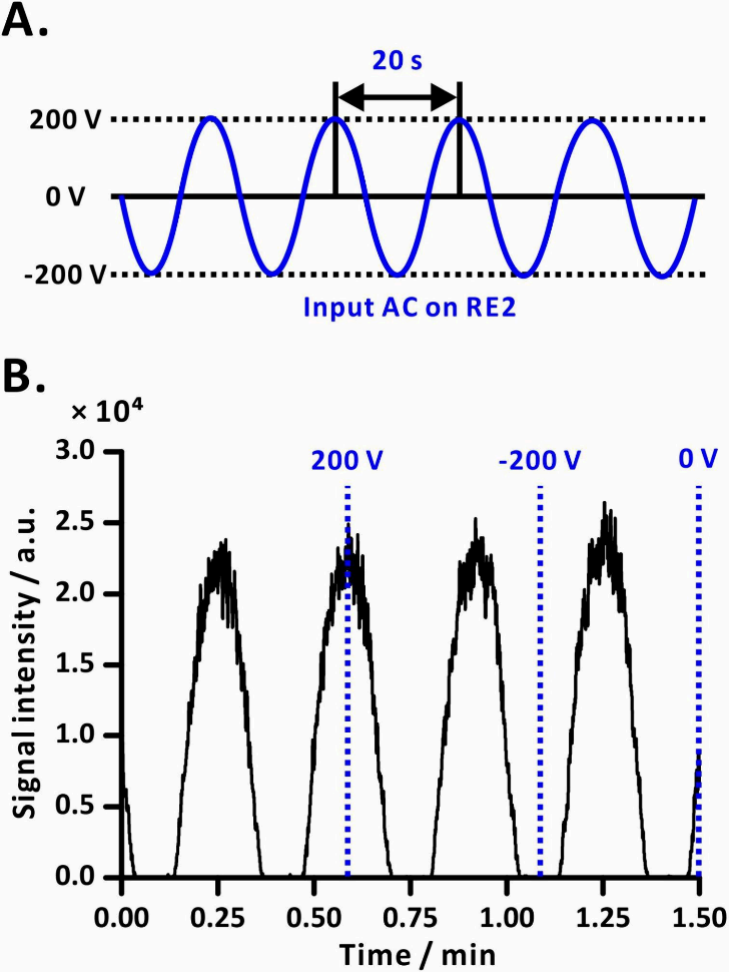
AC waveform applied to the RE2 (A) and the corresponding ion current of acetaminophen (*m*/*z* 152) recorded by QQQ-MS (B). The solid blue line represents the AC voltage input signal, while the dotted line corresponds to the output signal for that particular input voltage value.

The particle trajectory simulation results (Figure 3), obtained using SIMION, are in agreement with the electric field simulation results (Figure 2), obtained using COMSOL. Trajectories of particles with diameters of 1 nm (Figure S3A,C,E), 1 μm (Figure 3), and 100 μm (Figure S3B,D,F) were investigated. The starting position of the particles is set to be the tip of the ESI capillary, which is a reasonable simplification, at least for the large particles. For all three particle sizes, when 0 V is applied to the RE2, particle trajectories are dispersed around the MS inlet due to the lack of focusing effect from the RE2 (Figures 3A and S3A,B). This leads to a decrease in MS signal intensity (Figure 4B, approximately one-half the height of the peak leading and trailing edges). However, when 200 V is applied to the RE2, the particle trajectories converge near the MS inlet (Figures 3B and S3C,D), which is consistent with the convergence of electric field streamlines in the COMSOL simulation result (Figure 2B). This leads to a high MS signal intensity (Figure 4B, wave maxima). On the other hand, when −200 V is applied to the RE2, the particle propagation is clearly blocked by the electric field, which is consistent with the findings made in the COMSOL simulations; specifically, in the result of −200 V COMSOL simulation (Figure 2C), the black lines with arrows, representing the electric field streamlines, point in the opposite direction with respect to the MS inlet. This retarding force decreases the probability of ions and charged droplets entering the MS inlet. Additionally, this reversal of streamline directions can lead to disordered trajectories, particularly for the particles with larger masses and charges (Figures 3C and S3F). For the particles with smaller masses and charges, such as 1 nm diameter particles (Figure S3E), the electric field can block these particles entirely, so that they would not enter the MS inlet (Figure 4B, wave minima).

There are two practical implications of the simulation results obtained for 200 V applied to the RE2 (Figures 2B and 3B). First, in the region between the ESI needle and the MS inlet, the RE2 generates a gradually and uniformly decreasing electric field, potentially aiding ions and other charged droplets to enter the MS inlet, thereby compensating for the loss of ion transfer efficiency caused by the relatively long distance from the ESI needle to the MS inlet. Second, it is observed that the black lines with arrows in Figure 2B, which represent the electric field streamlines, noticeably converge near the hole in the RE2. This convergence may enhance charged particle transfer efficiency to the MS inlet due to the focusing effect.

### Application of the Developed System in Quantitative Analysis by QQQ-MS

In the first experiment, we modulated the ion current recorded by the QQQ-MS utilizing the ESI source with the three REs placed between the electrospray emitter and the MS inlet (Figure 1A). By applying a 0.05 Hz AC electric field (Figures 4A), periodic wave-like features were generated (Figure 4B). Following demodulation of the obtained MS wave-like ion currents with FFT, we found that the FFT magnitude, representing the amplitude of the frequency-domain signal, correlates with the analyte concentration (Figure S4). Relating the magnitude of the signal with concentration provides another approach for quantitative analysis.

It is worth noting that the ion current signal should ideally be at the highest value when the AC voltage on the RE2 crosses 200 V. However, we found a valley-like pattern formed at the top of the peak (Figure S5B). For this reason, we applied a positive DC voltage to the RE3 to stabilize the analyzed ions after they pass through the AC electric field region. Nevertheless, we noticed that too high of a positive voltage, applied to the RE3, results in severe suppression of the ion signal (Figure S6, *t* = 0.25–1.25 min). Thus, we chose to set the DC voltage on the RE3 to a relatively low value of 10 V.

Several parameters were optimized in order to acquire a steady MS ion current, including the distance from the RE3 to the MS inlet (Figure S7A,B), the drying gas flow rate (Figure S7C,D), the ESI voltage (Figure S7E,F), as well as the DL temperature (Figure S7G,H). Although the distance from the RE3 to the MS inlet of ∼10 mm (Figure S8A), and the drying gas flow rate of 0 L min^–1^ (Figure S8C), show the highest signal intensity and FFT magnitude, the resulting ion currents are very unstable (Figure S8A,C). Therefore, we selected ∼12.5 mm (Figure S8B) and 3 L min^–1^ (Figure S8D) as the optimum distance from the RE3 to the MS inlet and the drying gas flow rate, respectively. The ESI voltage of 4.0 kV and the DL temperature 150 °C have been chosen because of the high and stable signals recorded with these settings.

**Figure 5:**
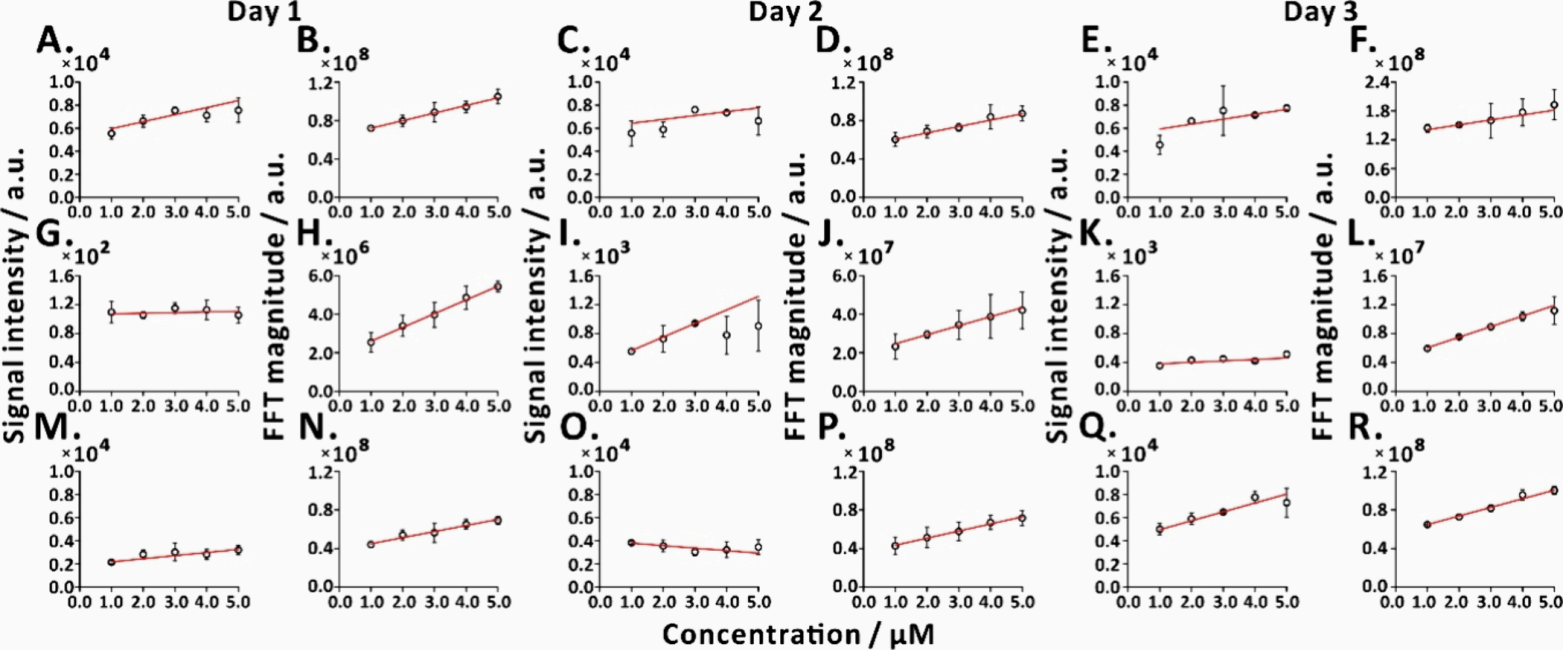
Calibration plots of acetaminophen (*m*/*z* 152), alanine (*m*/*z* 90), and lysine (*m*/*z* 147) obtained in 3 days. The red lines refer to the fitted functions (cf., Table S1). Quantification of acetaminophen [(A) day 1, (C) day 2, and (E) day 3, without the modulation of AC and FFT; (B) day 1, (D) day 2, and (F) day 3, with the modulation of 0.05 Hz AC and FFT]; quantification of alanine [(G) day 1, (I) day 2, and (K) day 3, without the modulation of AC and FFT; (H) day 1, (J) day 2, and (L) day 3, with the modulation of 0.05 Hz AC and FFT]; and quantification of lysine [(M) day 1, (O) day 2, and (Q) day 3, without the modulation of AC and FFT; (N) day 1, (P) day 2, and (R) day 3, with the modulation of 0.05 Hz AC and FFT]. The FFT magnitude was measured at frequency 0.0416 Hz that corresponds to the pattern in the raw QQQ-MS ion currents. The error bars represent standard deviation (*n* = 3).

The experiments involving three different analytes were performed on 3 days (Figure 5). At the same distance, the quantitative goodness-of-fit (*R*^2^) was improved under AC electric field modulation, as compared to the control experiment without AC modulation applied (Table S1). Without AC modulation and FFT, the *R*^2^ values for acetaminophen, alanine, and lysine were in the ranges of 0.2796–0.6934, 0.0532–0.9590, and 0.6865–0.9127, respectively. With AC modulation and FFT, the *R*^2^ values for acetaminophen, alanine, and lysine were in the ranges of 0.8652–0.9959, 0.9786–0.9972, and 0.9824–0.9944, respectively. This enhancement of *R*^2^ values may be contributed by the focusing effect produced by the 200 V modulation applied to the RE2, which stabilizes the trajectories of particles entering the MS inlet and reduces particle loss. Therefore, this experimental outcome highlights the ability of the focusing electric field to enhance particle transmission efficiency, potentially aiding in more precise quantitative analysis.

### Application of the Developed System in Qualitative Analysis by Q-TOF-MS

In order to expand the applications of AC field modulation of electrospray plume, we further combined this method with a high-resolution mass spectrometer (Q-TOF-MS). In this case, the purpose was to enhance qualitative analysis by precisely controlling *m*/*z* drifts of a calibration standard. The experimental setup used here resembled the one used previously with QQQ-MS. However, a nanoESI emitter was placed between the RE3 and Q-TOF-MS orifices in the direction perpendicular to the direction of the ESI emitter (Figure 1B).

In order to reduce the mutual interference between the two sprays, resulting from superposition of the nanoESI and ESI signals, we optimized several parameters. The distance between the RE3 and MS inlet (Figures S9), nanoESI flow rate (related to the pressure applied by the hydrodynamic pump, Figure S9; for the relationship between the applied pressure and flow rate, see Figure S10), ESI flow rate (Figure S11), drying gas flow rate (Figure S12), nanoESI voltage (Figure S13), ESI voltage (Figure S14), and DL temperature (Figure S15) were optimized. Each optimization experiment was divided into three stages: (A) reference signal with standard ESI without AC modulation (*t* = 0–0.5 min); (B) standard ESI with AC modulation (*t* = 0.5–1.5 min); and (C) applying voltage and pressure to the nanoESI electrolyte solution vial (*t* = 1.5–2.5 min). It can be noticed that the distance from the RE3 to the Q-TOF-MS orifice has a considerable impact on the signal of the AC-modulated ESI–MS pattern, especially when the nanoESI voltage and pressure are turned on at *t* = 1.5 min (Figure S9, red lines). This effect is most likely attributed to the fact that, when the ESI capillary is farther away from the orifice, the nanoESI plume enters the MS inlet predominantly. This diminishes the probability that ESI plume droplets and ions enter the MS inlet because of the electrostatic repulsion from positively charged nanoESI droplets and ions. At *t* = 1.5–2.5 min, the signals from the two sprays can be recorded for distances of ∼10 and ∼12.5 mm (Figure S9).

It is worth noting that there is a significant intensity difference between the resulting nanoESI and ESI signals at the distances of ∼10 and ∼12.5 mm. Although there is only a slight difference between the nanoESI signals at ∼10 and ∼12.5 mm distances, the corresponding AC-modulated ESI–MS ion current signals at ∼10 mm are higher than those at ∼12.5 mm. Therefore, we have finally decided to use the distance of ∼10 mm. For the pressure value of the nanoESI, ∼47 kPa was chosen because it yields a high and stable nanoESI–MS signal (Figure S9, red lines). The flow rate of the ESI sample does not have much effect on the corresponding ESI–MS signal (Figure S11, black lines). In fact, it was previously observed that, above a certain flow rate threshold, the ESI–MS signal intensity saturates.^39^ However, as the ESI sample flow rate increases, the nanoESI signal is dramatically suppressed (Figure S11, red lines). This may be due to the possibility that the increase in the ESI sample flow rate impacts the movement of nanodroplets and ions from the nanoESI emitter to the MS inlet. Alternatively, the large amount of solvent accompanying the high flow rate supplied by the ESI emitter may dilute the analyte present in nanoESI nanodroplets. For the flow rate value of the drying gas of 3.0 L min^–1^, the voltage value of the nanoESI and ESI of 4.0 kV, and the temperature value of the DL of 200 °C, were chosen because they yield a high and stable signal (Figures S12–S15).

**Figure 6:**
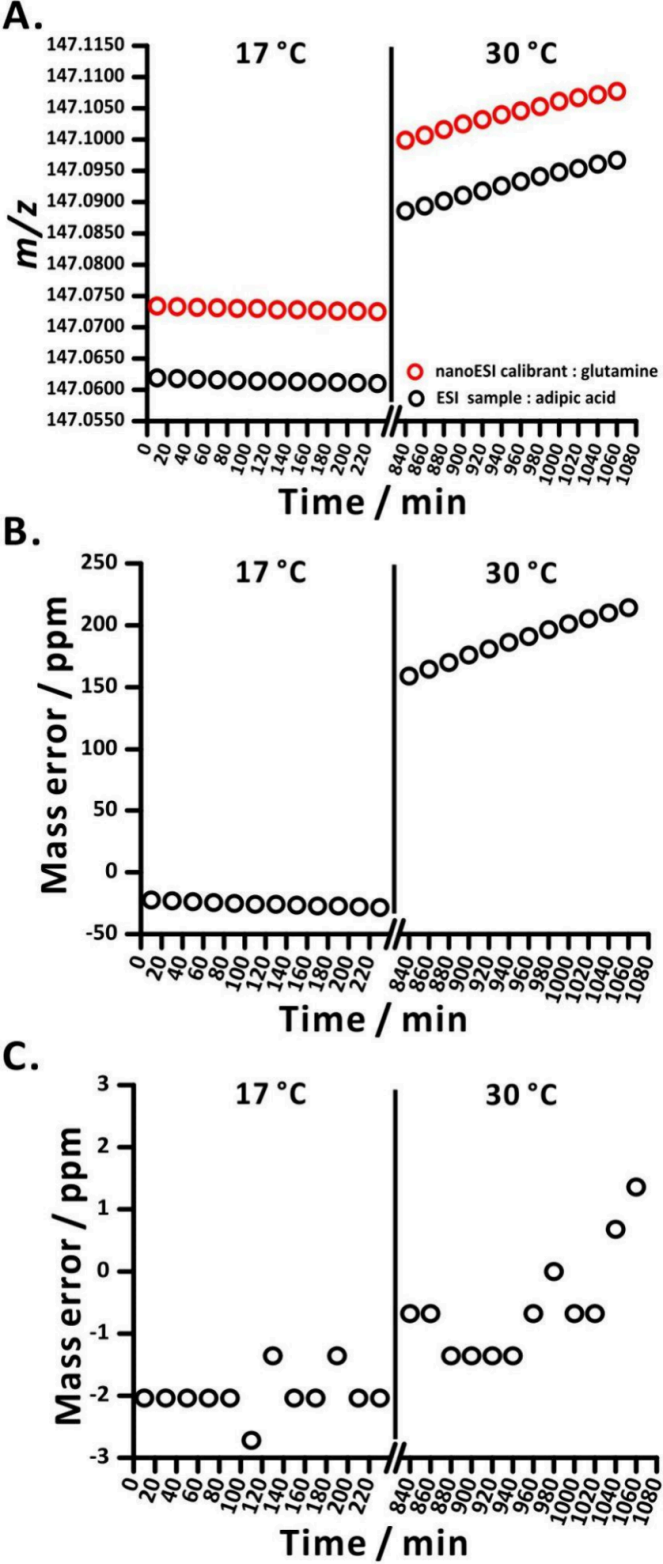
Q-TOF experiment designed to illustrate mass tracking in the presence of changing temperature. The first stage of this experiment was performed at a room temperature of 17 °C, while the second stage (after a break) was performed at a room temperature of 30 °C. The flight tube temperature control was switched off, and the mass calibration was carried out before the start, at 17 °C. (A) The *m*/*z* varies with increasing temperature. Red markers refer to the calibrant (glutamine) supplied by nanoESI, while black markers refer to the analyte (adipic acid) supplied by ESI. (B) Mass error of adipic acid without correction against glutamine standard over time with rising temperature. (C) Mass error of adipic acid corrected against glutamine standard over time with rising temperature.

For the purpose of investigating the impact of environmental temperature on instrument signal drift, we disabled the flight tube temperature controller device within the Q-TOF instrument. This was done to stop the warming process to reach the default temperature of 42 °C. Before starting the 4 h low temperature experiment, the following temperature procedure was performed to ensure that the temperature of the flight tube is similar to the environmental temperature. First, the environmental temperature was adjusted to 17 °C via the air conditioner, and kept at this level for about 2 days, so as to cool the flight tube to slightly above the environmental temperature (22 °C). Subsequently, the mass calibration was performed. After completing the 4 h low-temperature experiment (Figure 6A, before the break symbol), we raised the environmental temperature to 30 °C, and waited for ∼10 h until the flight tube temperature was close to the environmental temperature (28 °C). At that point, we started the 4 h high-temperature experiment (Figure 6A, after the break symbol). Please note that we purposely did not recalibrate the instrument right before the 30 °C experiments. Such recalibration would certainly improve mass accuracy at the beginning of this experiment stage. However, due to the inherent mass drift, caused by continuous temperature increase, it would not eliminate the huge mass error during the experiment. As expected, the collected data indicate a consistent ascending trend in the *m*/*z* of the calibrant (delivered by nanoESI) over time after the temperature increase, which aligns well with the *m*/*z* increase of the analyte (delivered by modulated ESI; Figure 6A). Please note that the time from switching the temperature to 30 °C and the continuation of the experiment was only ∼10 h. Thus, the innermost parts of the instrument probably did not reach the equilibrium temperature, which explains why the mass values kept on drifting.

To further investigate whether this device can effectively correct instrument drift to enhance mass accuracy, we calculated the mass error of adipic acid directly from the collected *m*/*z* data (without calibrant correction) and after recalibration, using the *m*/*z* of the calibrant glutamine, delivered by the nanoESI emitter (see the Experimental Section). As expected, without the mass correction (Figure 6B), the mass error increases with rising temperature, by approximately 100 ppm over a 4 h period. However, with the mass correction, the mass error value is significantly reduced (Figure 6C). This comparison demonstrates that by AC modulation of the ESI, alternating the introduction of sample and calibration solutions into the MS, mass tracking and recalibration can be successfully achieved.

In contrast to the previously disclosed dual-spray systems, used for mass calibration, employing the proposed device addresses three operational problems. First, the volume constraints imposed by separating the two sprays for sampling with a mechanical barrier are addressed. The nanoESI emitter is placed close to the MS orifice allowing for efficient delivery of calibrant ions. Second, the disclosed device also alleviates the challenges associated with the unreliable operation of mechanical actuation, such as liquid accumulation and requirement for frequent maintenance. Third, it achieves stable spray formation by eliminating the need to rapidly switch high voltages between two sprayers. For example, when using the electronic alternating switching method to dynamically control the sprayers, it is necessary to consider the possibility of residual liquid accumulation at the emitter tip when no voltage is applied because this could affect the stability of the spray once the voltage is turned on.

## Conclusions

We have demonstrated two ways for MS method calibration using alternating electric fields to modulate the electrospray plume and demodulation of the resulting MS ion currents using a simple mathematical treatment. Employing an ESI source with three REs, one receiving a sine electric signal, and, optionally, a nanoESI emitter between the ring electrodes and the mass spectrometer’s orifice, we observed wave-like features in recorded ion currents. In the first variant (QQQ mass analyzer), ion currents undergo FFT data treatment, which is followed by correlation of FFT magnitudes with analyte concentrations to produce calibration plots. In the second variant (Q-TOF mass analyzer), the mass spectra recorded at the analyte ion current maxima are mass-checked using the *m*/*z* value of internal standard (injected via nanoESI emitter), which appears predominantly in the time intervals corresponding to the analyte ion current minima. Please note that the current version of the system is suitable for direct infusion/shotgun analysis. When coupling this system with LC, the modulation frequency certainly has to be increased. Although, in this study, we used a single calibrant for mass tracking, in principle, it should be possible to inject a calibrant mixture for full mass calibration. It is also appealing to apply the developed system for analysis of a broader range of analytes and samples. This early demonstration of the proposed analytical method uses an on-axis electrospray emitter without nebulization. In the future, the setup can be upgraded using an off-axis electrospray emitter with nebulization in order to improve the method’s sensitivity further.

## Supporting Information Available

Additional table (S1; calibration equations); additional figures (S1–S15; vertical position of the ESI capillary; schematic of electronic circuit; SIMION simulation; wave-like features generated by modulating the electric field; comparison of the results obtained with the RE3 with and without DC voltage; optimization of the RE3 voltage; bar plots from optimization; optimization of the distance between the RE3 and the sampling cone of the QQQ-MS; optimization of the distance from the RE3 to the Q-TOF-MS inlet and the pressure applied to the vial with nanoESI electrolyte solution; relationship between flow rate and pressure applied to the nanoESI electrolyte solution vial; optimization of the ESI flow rate; optimization of the drying gas flow rate; optimization of the nanoESI voltage; optimization of the ESI voltage; and optimization of the DL temperature); and computer code
