# Supplementary material for: Electric Field-Modulated Electrospray Ionization Mass Spectrometry for Quantity Calibration and Mass Tracking
Source: J Am Soc Mass Spectrom. 2024 May 24;35(9):2064–72. doi: 10.1021/jasms.4c00091 (PMC11378279; doi:10.1021/jasms.4c00091)
Supplement: Supplementary file 1 — js4c00091_si_001.pdf [file js4c00091_si_001.pdf]

SUPPORTING INFORMATION

Electric Field-Modulated Electrospray Ionization Mass Spectrometry for Quantity Calibration and Mass Tracking

Pin-Chieh Hsu, Pawel L. Urban*

Department of Chemistry, National Tsing Hua University

101, Section 2, Kuang-Fu Rd., Hsinchu, 300044, Taiwan

* Corresponding author:

P.L. Urban (urban@mx.nthu.edu.tw)

ADDITIONAL TABLE

Table S1. Calibration equations for various tested analytes (n = 3). The analytes were dissolved in 25% (v/v) methanol in water. Concentration unit: μM. The RE3 potential in the control experiment: 0 V.

Compound (concentration range)		without AC and FFT calibration equation, R^2	with AC and FFT calibration equation, R^2
Acetaminophen (1.0 – 5.0 μM)	**Day 1**	$I = (6.09 \times 10^2 \pm 2.35 \times 10^2)C + (5.35 \times 10^3 \pm 0.68 \times 10^3)$ $R^2 = 0.6911$	$M = (7.89 \times 10^6 \pm 0.29 \times 10^6)C + (6.41 \times 10^7 \pm 0.07 \times 10^7)$ $R^2 = 0.9959$
	**Day 2**	$I = (3.33 \times 10^2 \pm 3.09 \times 10^2)C + (6.10 \times 10^3 \pm 1.13 \times 10^3)$ $R^2 = 0.2796$	$M = (6.63 \times 10^6 \pm 0.61 \times 10^6)C + (5.39 \times 10^7 \pm 0.18 \times 10^7)$ $R^2 = 0.9756$
	**Day 3**	$I = (4.27 \times 10^2 \pm 1.35 \times 10^2)C + (5.50 \times 10^3 \pm 0.52 \times 10^3)$ $R^2 = 0.6934$	$M = (1.02 \times 10^7 \pm 0.20 \times 10^7)C + (1.31 \times 10^8 \pm 0.04 \times 10^8)$ $R^2 = 0.8652$
Alanine (1.0 – 5.0 μM)	**Day 1**	$I = (8.88 \times 10^{-1} \pm 21.6 \times 10^{-1})C + (1.06 \times 10^2 \pm 0.06 \times 10^2)$ $R^2 = 0.0532$	$M = (7.14 \times 10^5 \pm 0.22 \times 10^5)C + (1.89 \times 10^6 \pm 0.09 \times 10^6)$ $R^2 = 0.9972$
	**Day 2**	$I = (1.88 \times 10^2 \pm 0.24 \times 10^2)C + (3.74 \times 10^2 \pm 0.63 \times 10^2)$ $R^2 = 0.9590$	$M = (4.69 \times 10^6 \pm 0.40 \times 10^6)C + (1.99 \times 10^7 \pm 0.09 \times 10^7)$ $R^2 = 0.9786$
	**Day 3**	$I = (2.00 \times 10^1 \pm 1.32 \times 10^1)C + (3.60 \times 10^2 \pm 0.44 \times 10^2)$ $R^2 = 0.4350$	$M = (1.47 \times 10^6 \pm 0.06 \times 10^6)C + (4.55 \times 10^6 \pm 0.12 \times 10^6)$ $R^2 = 0.9953$
Lysine (1.0 – 5.0 μM)	**Day 1**	$I = (2.69 \times 10^2 \pm 0.62 \times 10^2)C + (1.90 \times 10^3 \pm 0.13 \times 10^3)$ $R^2 = 0.8633$	$M = (6.31 \times 10^6 \pm 0.49 \times 10^6)C + (3.86 \times 10^7 \pm 0.16 \times 10^7)$ $R^2 = 0.9824$
	**Day 2**	$I = (-2.18 \times 10^2 \pm 0.85 \times 10^2)C + (4.01 \times 10^3 \pm 0.16 \times 10^3)$ $R^2 = 0.6865$	$M = (7.38 \times 10^6 \pm 0.43 \times 10^6)C + (3.60 \times 10^7 \pm 0.15 \times 10^7)$ $R^2 = 0.9899$
	**Day 3**	$I = (7.70 \times 10^2 \pm 1.38 \times 10^2)C + (4.19 \times 10^3 \pm 0.41 \times 10^3)$ $R^2 = 0.9127$	$M = (8.96 \times 10^6 \pm 0.39 \times 10^6)C + (5.58 \times 10^7 \pm 0.05 \times 10^7)$ $R^2 = 0.9944$

ADDITIONAL FIGURES

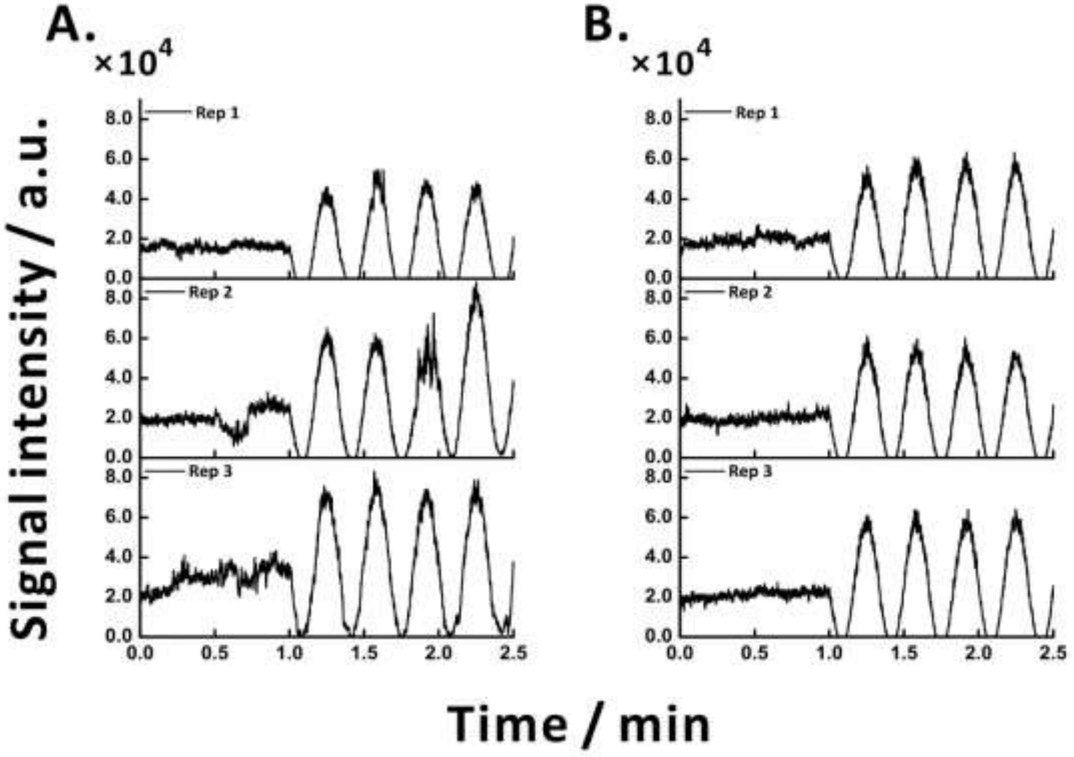

Figure S1. Vertical position of the ESI capillary from the center of MS inlet (A) ~ 0 mm; (B) higher ~ 1 mm. The sample was 5 μM of analyte dissolved in 25% (v/v) methanol in water. The flow rate was 40 μL min^{-1}. The QQQ-MS was operated in SIM mode (lysine *m/z* 147).

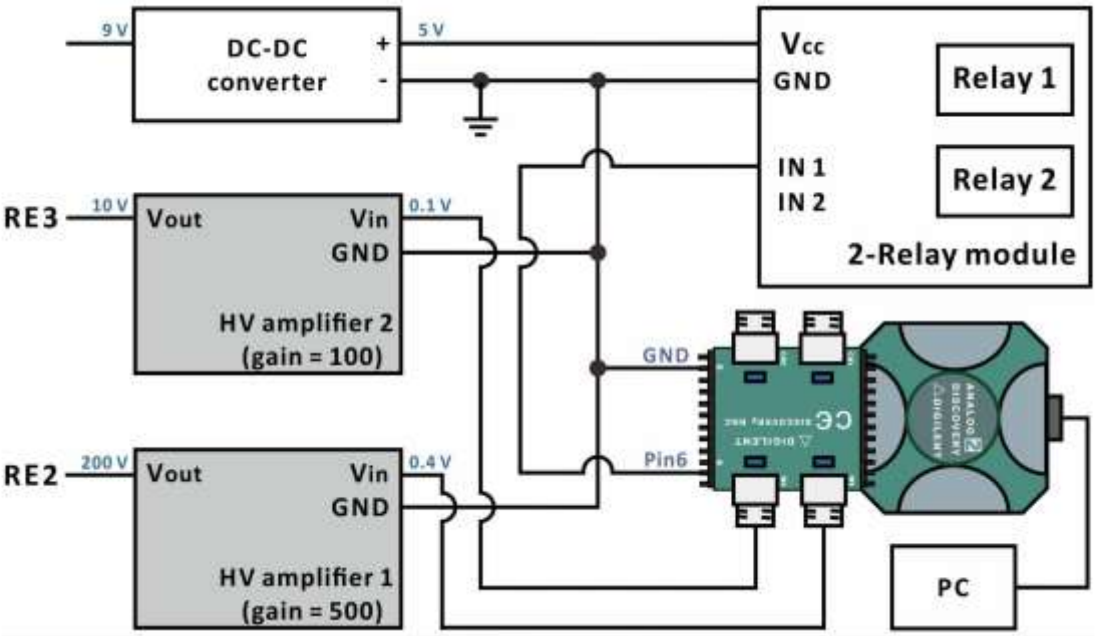

Figure S2. Schematic of the electronic circuit used to control electrospray plume with AC field.

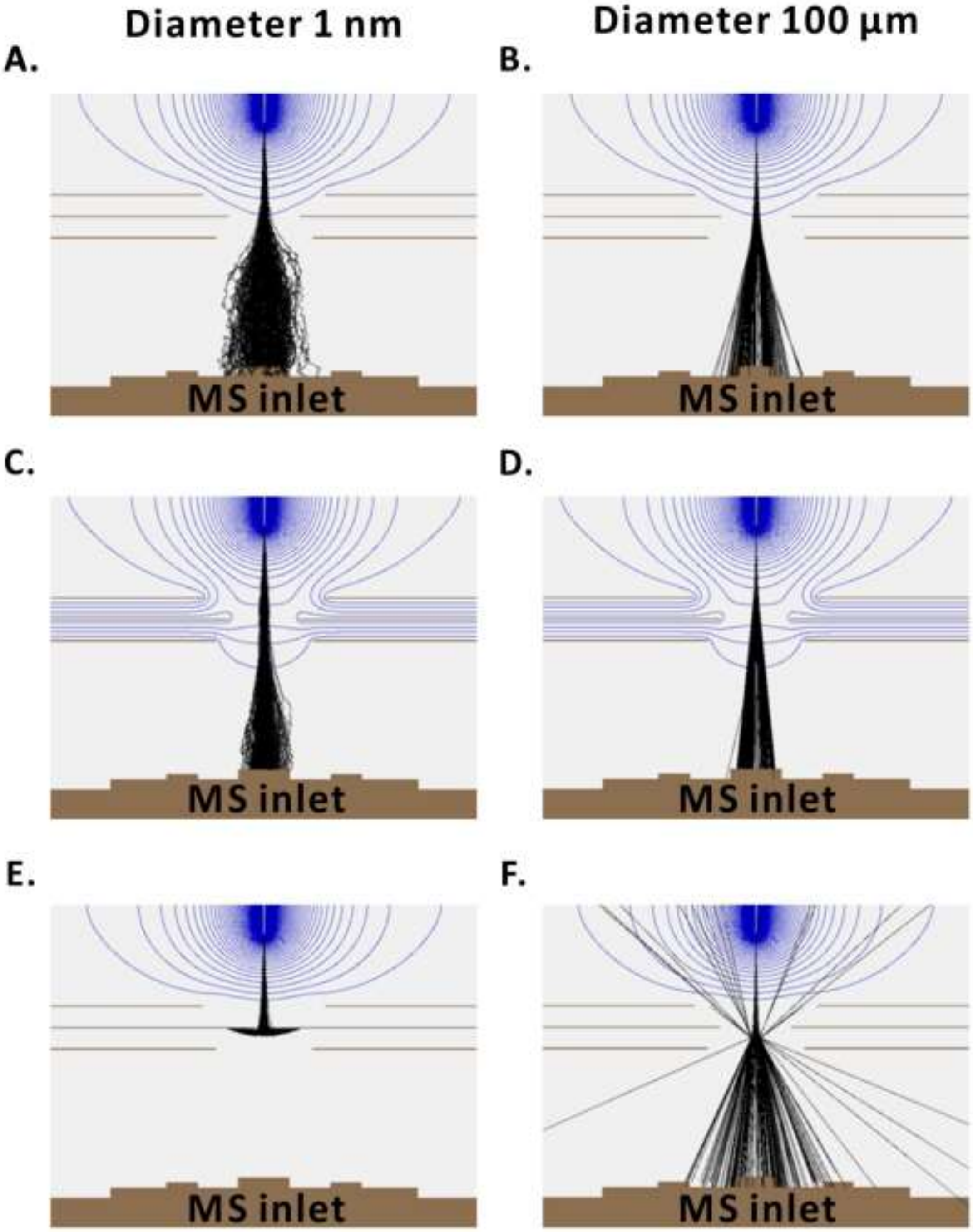

Figure S3. The SIMION simulation examines the trajectories of particles with different diameters under three different voltages applied to the RE2: (A) 1 nm diameter particles ($Q_{min} \approx 0.78$ e, $Q_{max} \approx 1.41$ e and $M_{min} \approx 2.49 \times 10^2$ u, $M_{max} \approx 3.14 \times 10^2$ u); (B) 100 µm diameter particles ($Q_{min} \approx 2.48 \times 10^7$ e, $Q_{max} \approx 4.46 \times 10^7$ e and $M_{min} \approx 2.50 \times 10^{17}$ u, $M_{max} \approx 3.15 \times 10^{17}$ u) when the RE2 is supplied with 0 V; (C) 1 nm diameter particles; (D) 100 µm diameter particles when the RE2 is supplied with 200 V; (E) 1 nm diameter particles; (F) 100 µm diameter particles when the RE2 is supplied with -200 V. The number of particles was 200.

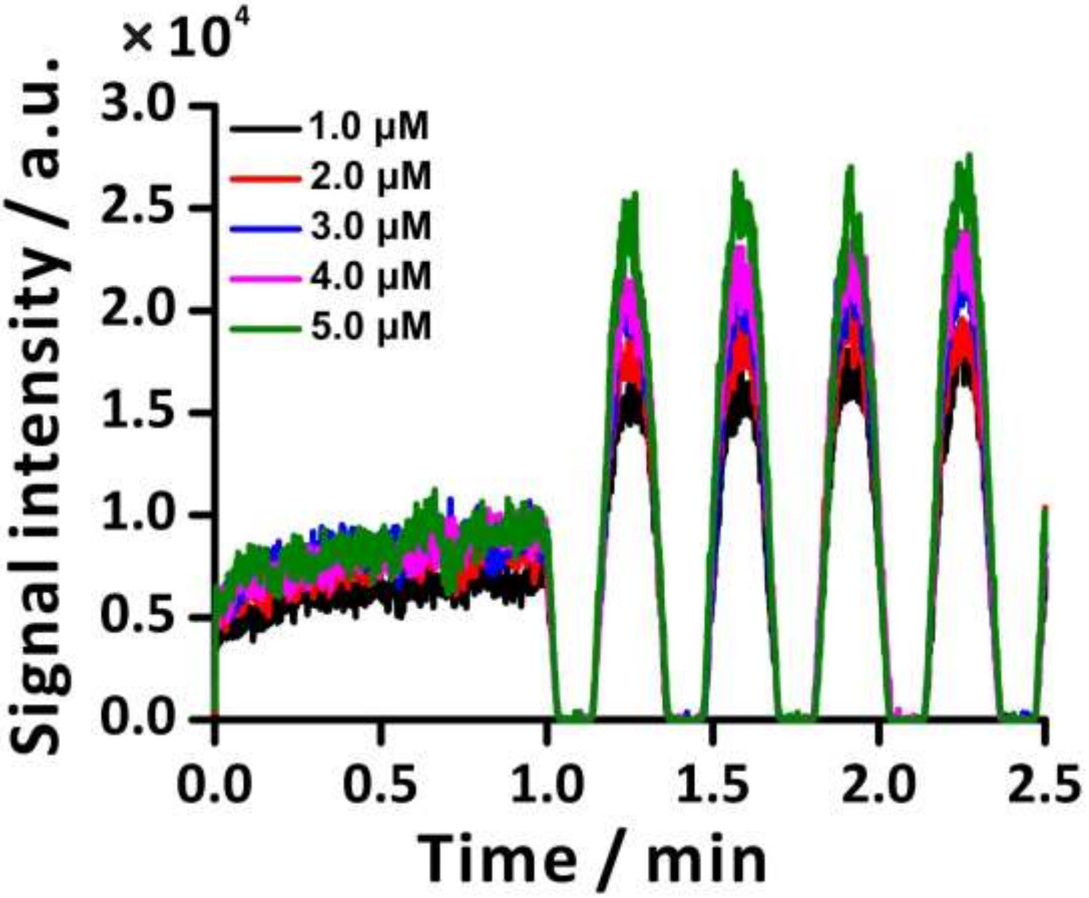

Figure S4. Wave-like features are generated by modulating the electric field with 0.05 Hz frequency. Each color refers to a different concentration of acetaminophen. Sequence: t = 0.00-0.50 min, turn on the ESI; t = 0.50-1.00 min, turn on the RE3 voltage; t = 1.00-2.50 min, turn on the RE2 AC voltage.

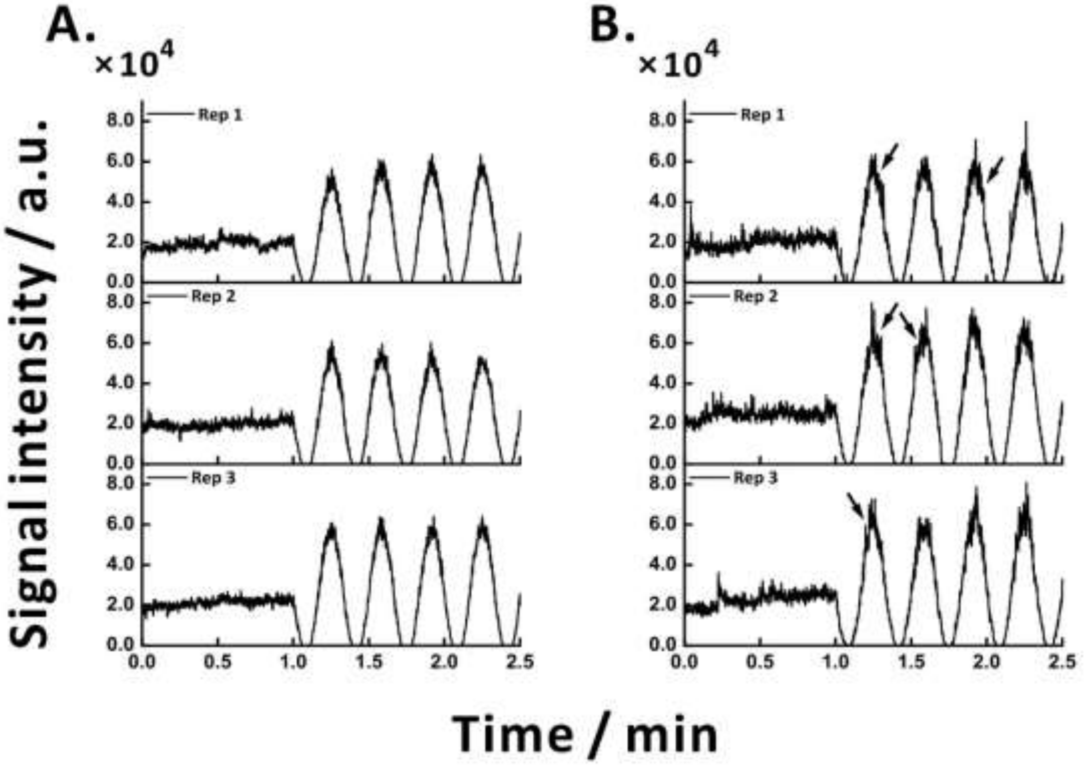

Figure S5. Comparison of the results obtained with the RE3 with and without DC voltage. (A) The RE3 with DC 10 V; (B) the RE3 without DC 10 V. The sample was 5 μM of analyte dissolved in 25% (v/v) methanol in water. The flow rate was 40 μL min^{-1}. The QQQ-MS was operated in SIM mode (lysine *m/z* 147). The arrows in (B) show the instabilities. The standard deviations of the signals in the 0-1 min region are (A) 1104 a.u. and (B) 2296 a.u.

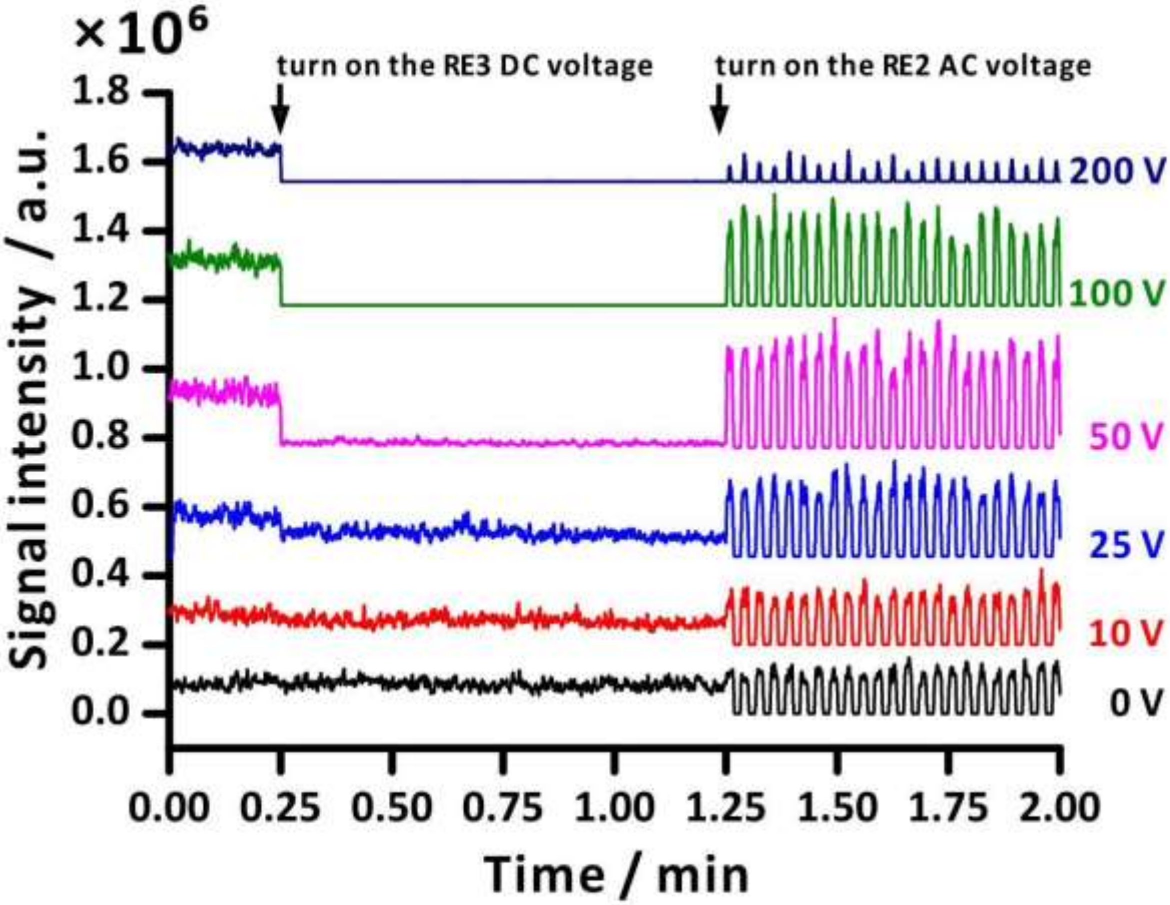

Figure S6. Optimization of the RE3 voltage. *t* = 0.00-0.25 min turn on the ESI, *t* = 0.25-1.25 min turn on the RE3 voltage, *t* = 1.25-2.00 min turn on the RE2 AC voltage. The sample was 5 µM of lysine dissolved in 25% (v/v) methanol in water. The flow rate was 40 µL min^{-1}. The QQQ-MS was operated in SIM mode (lysine *m/z* 147). The data traces have been shifted vertically for clarity.

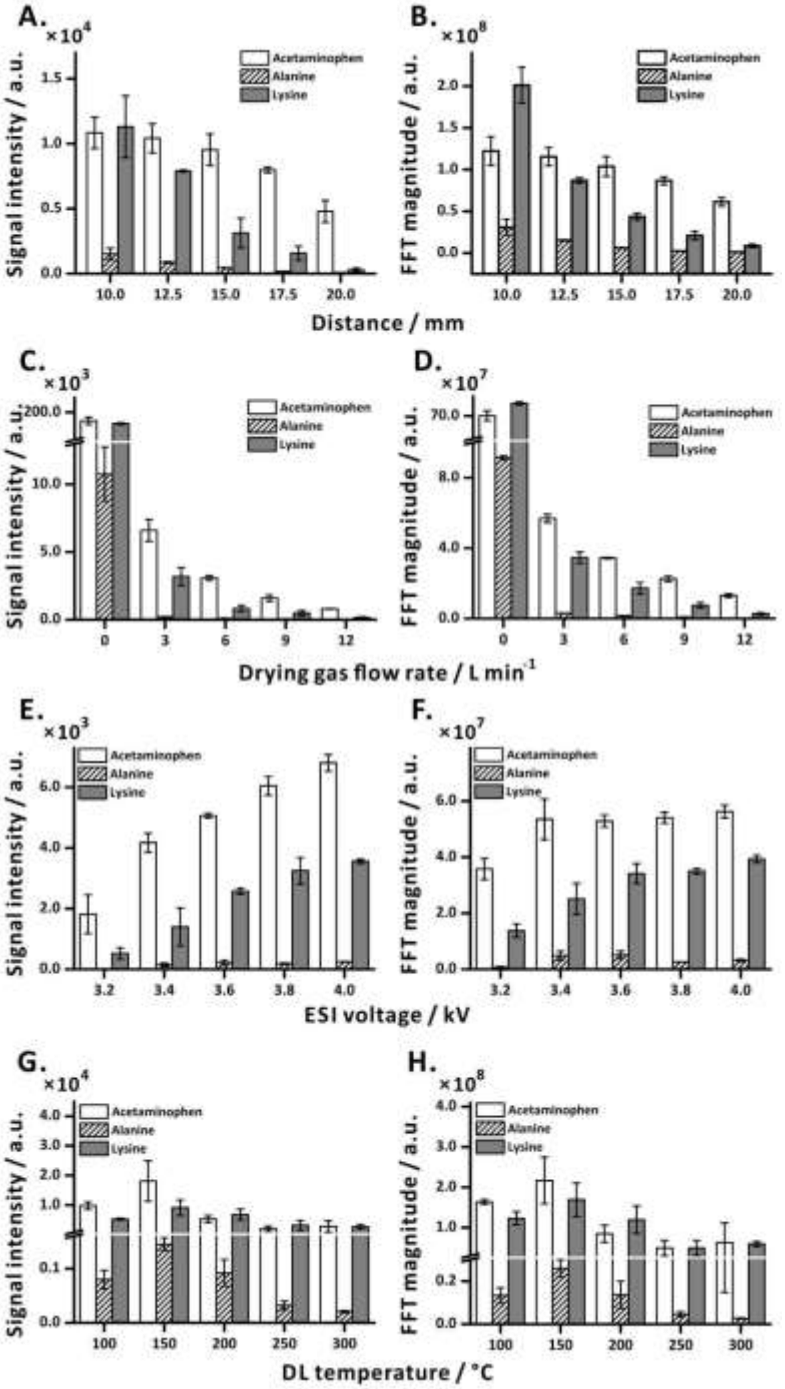

Figure S7. Bar plots representing (A) signal intensity and (B) FFT magnitude for the varied distance between the RE3 and the sampling cone of the QQQ-MS; (C) signal intensity and (D) FFT magnitude for the varied drying gas flow rate of the QQQ-MS; (E) signal intensity and (F) FFT magnitude for the varied ESI voltage of the QQQ-MS; (G) signal intensity and (H) FFT magnitude for the varied DL temperature of the QQQ-MS. The sample was 5 μM of analyte dissolved in 25% (v/v) methanol in water. The flow rate was 40 μL min^{-1}. The QQQ-MS was operated in SIM mode (acetaminophen *m/z* 152, alanine *m/z* 90, lysine *m/z* 147).

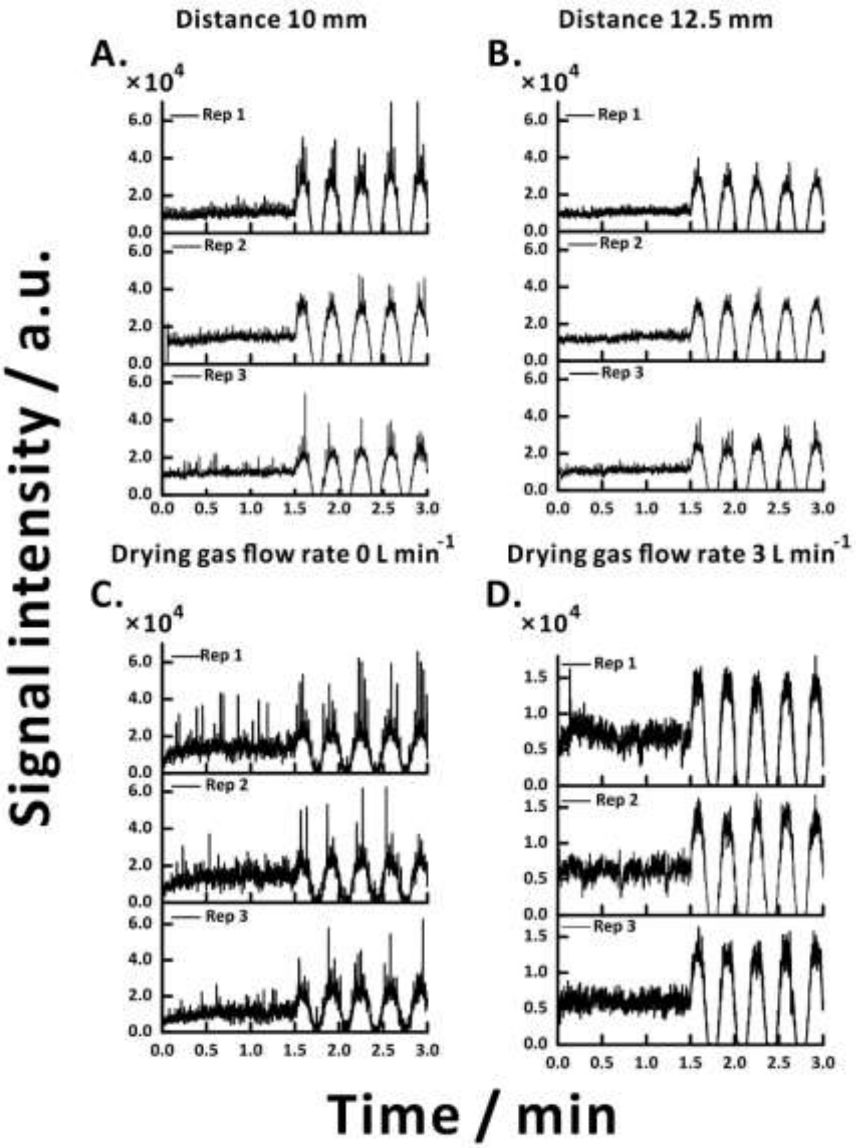

Figure S8. Optimization of the distance between the RE3 and the sampling cone of the QQQ-MS [(A) ~ 10 mm; (B) ~ 12.5 mm]; and QQQ-MS drying gas flow rate [(C) 0 L min^{-1}; (D) 3 L min^{-1}]. The sample was 5 µM of analyte dissolved in 25% (v/v) methanol in water. The flow rate was 40 µL min^{-1}. The QQQ-MS was operated in SIM mode (lysine *m/z* 147).

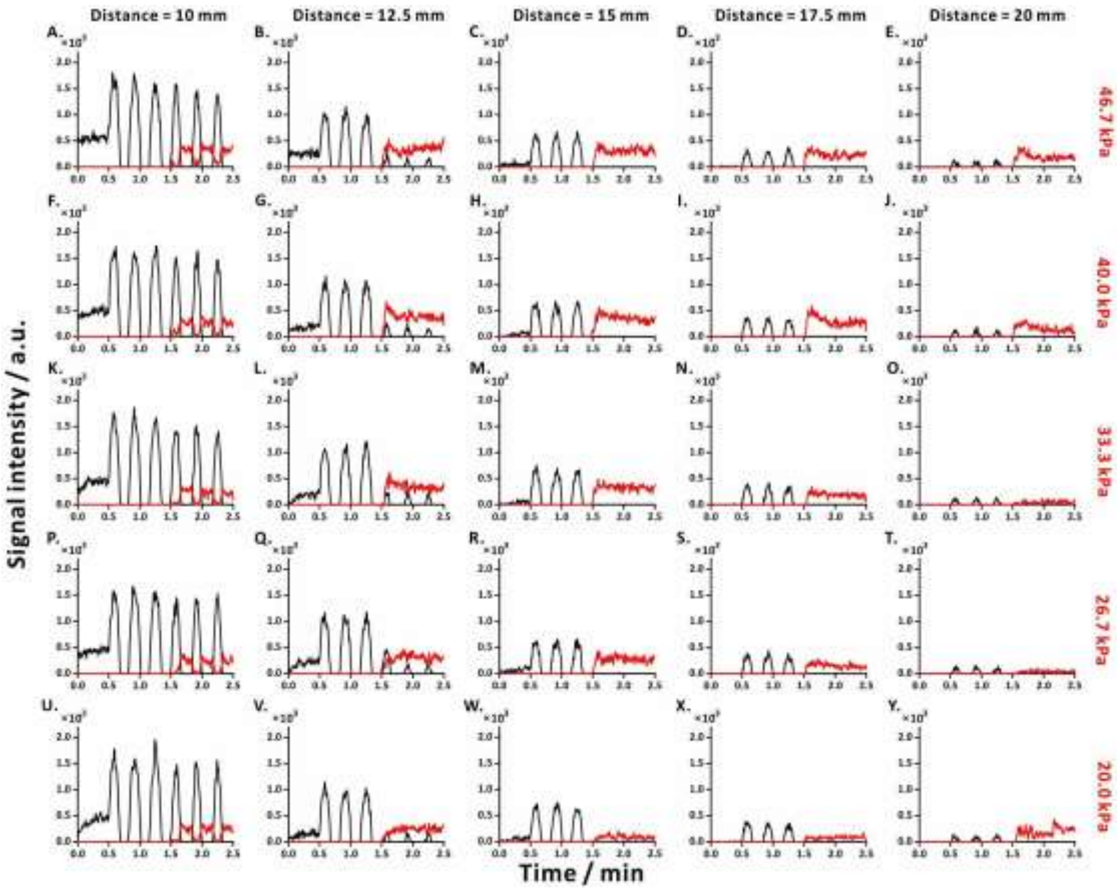

Figure S9. Optimization of the distance from the RE3 to the Q-TOF-MS inlet and the pressure applied to the vial with nanoESI electrolyte solution. The black line refers to the ESI plume modulated with AC (50 µM adipic acid, *m/z* 147.0652) while the red line refers to the nanoESI plume of a continuously sampled calibrant (5 µM L-glutamine, *m/z* 147.0764). Both the sample and calibrant were dissolved in 25% (v/v) methanol in water. The flow rate of ESI was 10 µL min^{-1}. The drying gas flow rate was 3.0 L min^{-1}. The voltage applied to ESI and nanoESI was 4.0 kV in both cases. The DL temperature was 200 °C. The Q-TOF-MS was operated in MS scan mode.

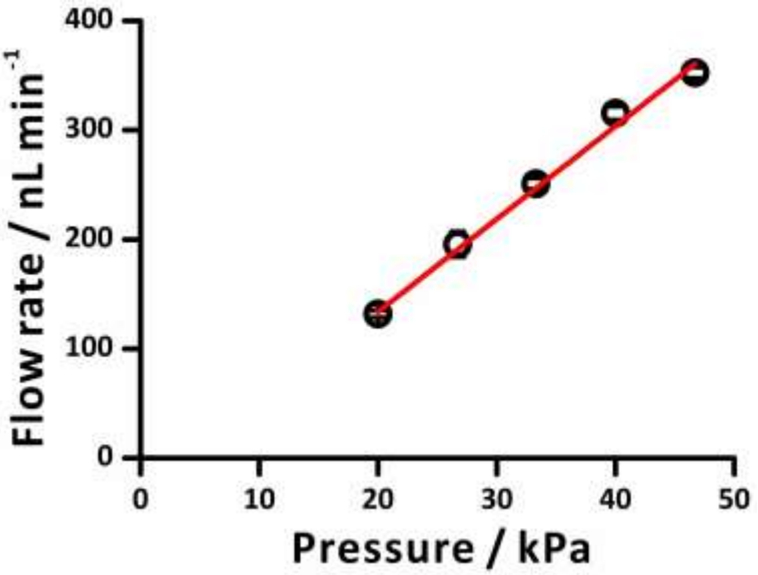

Figure S10. Relationship between flow rate and pressure applied to the nanoESI electrolyte solution vial. The solution was 25% (v/v) methanol in water. Calibration equation: $y = (8.49 \pm 0.33)\ x + (-3.61 \times 10^1 \pm 1.05 \times 10^1)$, $R^2 = 0.99415$.

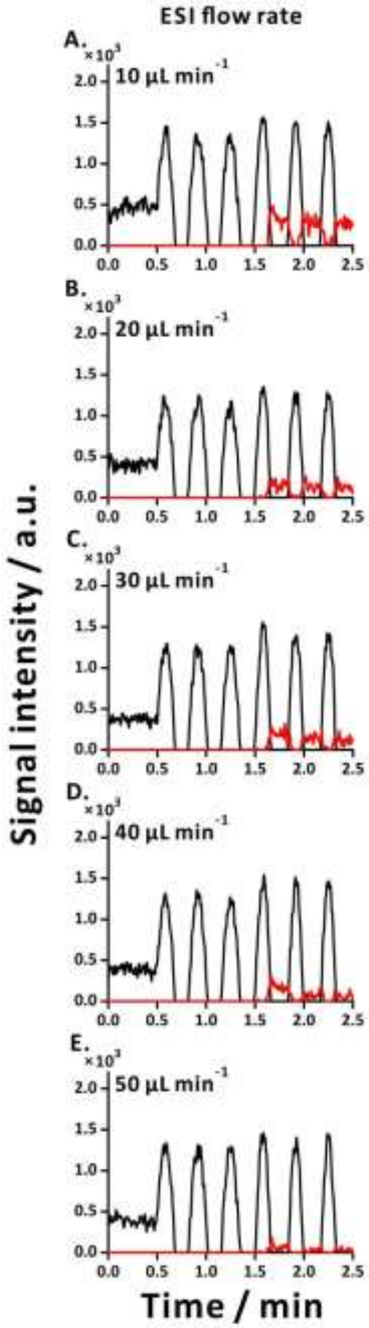

Figure S11. Optimization of the ESI flow rate: (A) 10 µL min^{-1}; (B) 20 µL min^{-1}; (C) 30 µL min^{-1}; (D) 40 µL min^{-1}; (E) 50 µL min^{-1}. The black line refers to the ESI plume modulated with AC (50 µM adipic acid, *m/z* 147.0652) while the red line refers to the nanoESI plume of a continuously sampled calibrant (5 µM L–glutamine, *m/z* 147.0764). Both the sample and calibrant were dissolved in 25% (v/v) methanol in water. The distance from the RE3 to the Q-TOF-MS inlet was ~ 10 mm. The pressure applied to the vial with nanoESI electrolyte solution was ~ 47 kPa. The drying gas flow rate was 3.0 L min^{-1}. The voltage applied to ESI and nanoESI both were 4.0 kV. The DL temperature was 200 °C. The Q-TOF-MS was operated in MS scan mode.

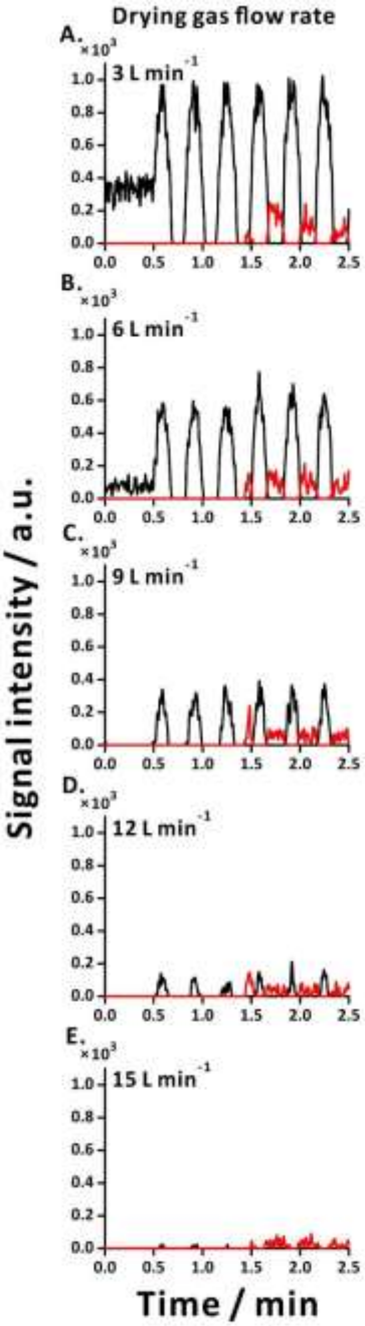

Figure S12. Optimization of the drying gas flow rate: (A) 3 L min^{-1}; (B) 6 L min^{-1}; (C) 9 L min^{-1}; (D) 12 L min^{-1}; (E) 15 L min^{-1}. The black line refers to the ESI plume modulated with AC (50 µM adipic acid, *m/z* 147.0652) while the red line refers to the nanoESI plume of a continuously sampled calibrant (5 µM L-glutamine, *m/z* 147.0764). Both the sample and calibrant were dissolved in 25% (v/v) methanol in water. The distance from the RE3 to the Q-TOF-MS inlet was ~ 10 mm. The pressure applied to the vial with nanoESI electrolyte solution was ~ 47 kPa. The flow rate of ESI was 10 µL min^{-1}. The voltage applied to ESI and nanoESI both were 4.0 kV. The DL temperature was 200 °C. The Q-TOF-MS was operated in MS scan mode.

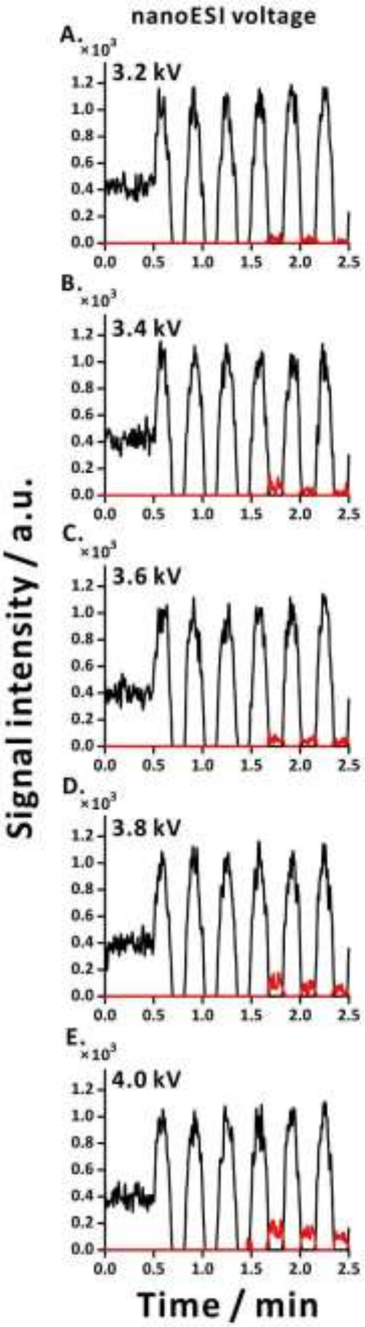

Figure S13. Optimization of the nanoESI voltage: (A) 3.2 kV; (B) 3.4 kV; (C) 3.6 kV; (D) 3.8 kV; (E) 4.0 kV. The black line refers to the ESI plume modulated with AC (50 µM adipic acid, *m/z* 147.0652) while the red line refers to the nanoESI plume of a continuously sampled calibrant (5 µM L-glutamine, *m/z* 147.0764). Both the sample and calibrant were dissolved in 25% (v/v) methanol in water. The distance from the RE3 to the Q-TOF-MS inlet was ~ 10 mm. The pressure applied to the vial with nanoESI electrolyte solution was ~ 47 kPa. The flow rate of ESI was 10 µL min^{-1}. The drying gas flow rate was 3.0 L min^{-1}. The voltage applied to ESI was 4.0 kV. The DL temperature was 200 °C. The Q-TOF-MS was operated in MS scan mode.

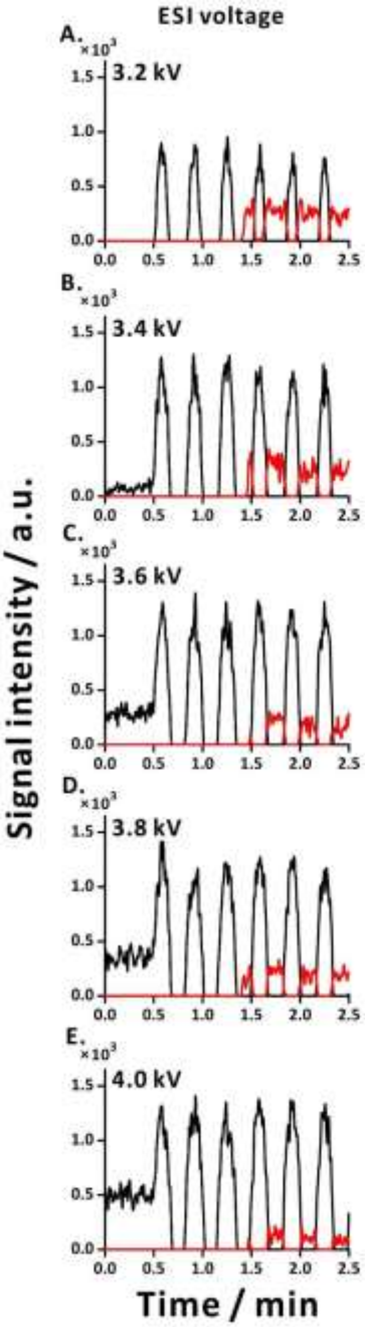

Figure S14. Optimization of the ESI voltage: (A) 3.2 kV; (B) 3.4 kV; (C) 3.6 kV; (D) 3.8 kV; (E) 4.0 kV. The black line refers to the ESI plume modulated with AC (50 µM adipic acid, *m/z* 147.0652) while the red line refers to the nanoESI plume of a continuously sampled calibrant (5 µM L-glutamine, *m/z* 147.0764). Both the sample and calibrant were dissolved in 25% (v/v) methanol in water. The distance from the RE3 to the Q-TOF-MS inlet was ~ 10 mm. The pressure applied to the vial with nanoESI electrolyte solution was ~ 47 kPa. The flow rate of ESI was 10 µL min^{-1}. The drying gas flow rate was 3.0 L min^{-1}. The voltage applied to nanoESI was 4.0 kV. The DL temperature was 200 °C. The Q-TOF-MS was operated in MS scan mode.

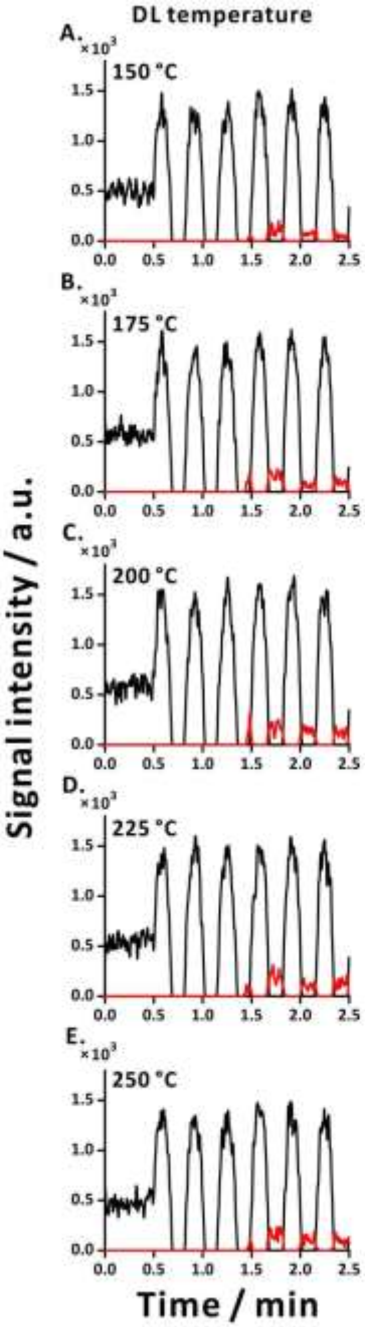

Figure S15. Optimization of the DL temperature: (A) 150 °C; (B) 175 °C; (C) 200 °C; (D) 225 °C; (E) 250 °C. The black line refers to the ESI plume modulated with AC (50 μM adipic acid, *m/z* 147.0652) while the red line refers to the nanoESI plume of a continuously sampled calibrant (5 μM L-glutamine, *m/z* 147.0764). Both the sample and calibrant were dissolved in 25% (v/v) methanol in water. The distance from the RE3 to the Q-TOF-MS inlet was ~ 10 mm. The pressure applied to the vial with nanoESI electrolyte solution was ~ 47 kPa. The flow rate of ESI was 10 μL min^{-1}. The drying gas flow rate was 3.0 L min^{-1}. The voltage applied to ESI and nanoESI both were 4.0 kV. The Q-TOF-MS was operated in MS scan mode.

COMPUTER CODE

Quantitative analysis by QQQ-MS: circuit control for the RE2 and RE3 (Arduino)

```
clear()
if(!('StaticIO' in this)) throw "Please open the StaticIO instrument";
if(!('Wavegen' in this) || !('Scope' in this)) throw "Please open a Scope
and a Wavegen instrument";
print("Running StaticIO script");
StaticIO.Channel0.Mode.text = "IOs";
StaticIO.Channel0.DIO6.Mode.text = "Button";
StaticIO.Channel0.DIO6.text = "1";
wait(0.1); % time in second
StaticIO.Channel0.DIO6.text = "0";
wait(30); % time in second
Wavegen.run();
Wavegen.Channel2.Mode.text = "Simple";
Wavegen.Channel2.Simple.Type.text = "DC";
Wavegen.Channel2.Simple.Offset.value = 0.1; % DC voltage in V
wait(30); % time in second
Wavegen.Channel1.Mode.text = "Simple";
Wavegen.Channel1.Simple.Type.text = "Sine"; % AC waveform
Wavegen.Channel1.Simple.Frequency.value = 0.05; % AC frequency in Hz
Wavegen.Channel1.Simple.Amplitude.value = 0.4; % AC amplitude in V
Wavegen.Channel1.Simple.Symmetry.value = 50; % Symmetry of AC in %
Wavegen.Channel1.Simple.Phase.value = 0;
Wavegen.Channel2.Mode.text = "Simple";
Wavegen.Channel2.Simple.Type.text = "DC"; % DC waveform
Wavegen.Channel2.Simple.Offset.value = 0.1; % DC voltage offset in V
wait(90); % time in second
Wavegen.stop();
```

Data processing of ion currents by FFT (Matlab)

```
clc;clear all;close all;
R1=readmatrix('MS data.xlsx','Sheet','conc 1 µM.','Range','B22508:B37514');
R2=readmatrix('MS data.xlsx','Sheet','conc 1 µM.','Range','B22508:B37514');
R3=readmatrix('MS data.xlsx','Sheet','conc 1 µM.','Range','B22508:B37514');
duration=150; %data acquisition time
n = length(B);
t=linspace(0,duration,n); % create a time point between 0 and duration that
matches the intensity data point (the interval is the event time)
Tevent = 0.004; %event time
yR1 = fft(R1); % replicate 1 time domain data transformed by FFT
yR2 = fft(R1); % replicate 2 time domain data transformed by FFT
yR3 = fft(R1); % replicate 3 time domain data transformed by FFT
fs = 1/Tevent;
fR1 = (0:length(yR1)-1)*fs/length(yR1);
% replicate 1 frequency vector corresponding to time domain data
fR2 = (0:length(yR2)-1)*fs/length(yR2);
% replicate 2 frequency vector corresponding to time domain data
fR3 = (0:length(yR3)-1)*fs/length(yR3);
% replicate 3 frequency vector corresponding to time domain data
fshift = (-n/2:n/2-1)*(fs/n); % move the zero-frequency component to the
center of the array and observe the spectrum of (-fs/2,fs/2)
yR1shift = fftshift(yR1);
yR2shift = fftshift(yR2);
yR3shift = fftshift(yR3);
plot(fshift,abs(yR1shift),'linewidth',2, 'Color',"#000000");
hold on
plot(fshift,abs(yR2shift),'linewidth',2, 'Color',"#FF00FF");
plot(fshift,abs(yR3shift),'linewidth',2, 'Color',"#0000FF");
xlabel('Frequency / Hz') % x-axis is the frequency in Hz
ylabel('FFT Magnitude / a.u.') % y-axis is the FFT magnitude in a.u.
axis([0 1 0 inf]) % axis start from x=0 y=0
hold off
```

Qualitative analysis by Q-TOF-MS: circuit control for the RE2 and RE3 (Arduino)

```
clear()
if(!('StaticIO' in this)) throw "Please open the StaticIO instrument";
if(!('Wavegen' in this) || !('Scope' in this)) throw "Please open a Scope
and a Wavegen instrument";
print("Running StaticIO script");
StaticIO.Channel0.Mode.text = "IOs";
StaticIO.Channel0.DIO6.Mode.text = "Button";
StaticIO.Channel0.DIO6.text = "1";
wait(0.1); % time in second
StaticIO.Channel0.DIO6.text = "0";
wait(30); % time in second
Wavegen.run();
Wavegen.Channel1.Mode.text = "Simple";
Wavegen.Channel1.Simple.Type.text = "Sine"; % AC waveform
Wavegen.Channel1.Simple.Frequency.value = 0.05; % AC frequency in Hz
Wavegen.Channel1.Simple.Amplitude.value = 0.4; % AC amplitude in V
Wavegen.Channel1.Simple.Symmetry.value = 50; % Symmetry of AC in %
Wavegen.Channel1.Simple.Phase.value = 0;
Wavegen.Channel2.Mode.text = "Simple";
Wavegen.Channel2.Simple.Type.text = "DC"; % DC waveform
Wavegen.Channel2.Simple.Offset.value = 0.1; % DC voltage offset in V
wait(120); % time in second
Wavegen.stop();
```
